# A comparative study on phytochemical analysis and biological properties of three varieties of *cannabis sativa L. seeds*

**DOI:** 10.1515/biol-2025-1211

**Published:** 2026-02-06

**Authors:** Rafik El-Mernissi, Naoual El Menyiy, Aziz Zouhri, Yahya El-Mernissi, Amira Metouekel, Farhan Siddique, Mohammed H. AL Mughram, Denekew Temesgen, Abdullah R. Alanzi, Mohammad Khalid, Hassan Amhamdi, Oualid Abboussi, Lhoussain Hajji

**Affiliations:** Faculty of Sciences, Bioactives and Environmental Health Laboratory, Moulay Ismail University, Meknes, 11201, Morocco; Faculty of Sciences, Physiology and Physiopathology Team, Genomic of Human Pathologies Research Centre, Mohammed V University, Rabat, Morocco; Laboratory of Pharmacology, National Agency for Medicinal and Aromatic Plants, Taounate, 34025, Morocco; Faculty of Science and Techniques, Applied Chemistry Research Unit, Abdelmalek Essaadi University, Al-Hoceima, Tetouan, 34025, Morocco; BOI R&D Laboratory, Bioval Ocean Indian Research and Innovation Company, 18 Rue Des Poivres Roses, 97419, La Possession, Reunion Island, France; Sorbonne Universities, University of Technology of Compiègne, EA 4297 TIMR, 60205, Compiègne Cedex, France; School of Pharmaceutical Science and Technology, Tianjin University, Tianjin, P.R. China; Department of Pharmaceutical Chemistry, College of Pharmacy, King Khalid University, 61421, Abha, Saudi Arabia; Department of Biology, Bahir Dar University, Bahir Dar, Ethiopia; Center for Research on Medicinal, Aromatic, and Poisonous Plants, DSR, King Saud University, 61421, Riyadh, Saudi Arabia; Department of Pharmaceutics, College of Pharmacy, King Khalid University, 61421, Abha, Saudi Arabia

**Keywords:** anti-nociceptive activity, antioxidant activity, anti-inflammatory activity, cannabis Sativa L seeds, HPLC-DAD

## Abstract

This study evaluated the antioxidant, anti-inflammatory, and analgesic properties of three varieties of *Cannabis sativa L.* seeds from Morocco, alongside their chemical compositions. High-Performance Liquid Chromatography with Diode Array Detection (HPLC-DAD) were employed for chemical analysis. Antioxidant activity was assessed using ABTS, TAC, and ferric reducing antioxidant power (FRAP) assays, while anti-inflammatory and analgesic effects were tested in animal models. Molecular docking targeted 5IKQ and 3RP8 enzymes based on HPLC-identified compounds. The hydroalcoholic extracts demonstrated appreciable levels of phenolics and flavonoids: total phenolic content (TPC) was 76.87 ± 0.24 mg GAE/g DW (Cric), 81.45 ± 1.37 mg GAE/g DW (Khard), and 84.96 ± 2.05 mg GAE/g DW (Beldiya), while total flavonoid content (TFC) was 3.34 ± 0.22 mg QE/g DW (Cric), 3.56 ± 0.07 mg QE/g DW (Khard), and 3.32 ± 0.12 mg QE/g DW (Beldiya).HPLC results revealed polyphenolic compounds, including Catechin, Quercetin, Ursolic acid, and Rosmarinic acid. The Beldiya variety showed the strongest antioxidant activity, with IC50 values of 0.12 ± 0.07 mg/mL (DPPH), 0.71 ± 0.01 mg/mL (ABTS), and 0.32 ± 0.04 mg/mL (FRAP). It also exhibited notable anti-inflammatory and analgesic effects at 300 mg/kg, comparable to aspirin and indomethacin. Molecular docking confirmed Quercetin, Catechin, and Rosmarinic acid as potent antioxidants, with Quercetin, Catechin, and Ursolic acid showing significant anti-inflammatory and analgesic potential. These findings underscore the therapeutic value of *Cannabis sativa* seeds for health applications.

## 1 Introduction

*Cannabis sativa* L. is an annual herbaceous species widely valued for its multipurpose applications, ranging from medicinal and therapeutic products to fiber and oilseed production. Considered among the earliest domesticated crops, it is generally believed to have originated in Asia [1], 2]. Taxonomically, *C. sativa* is broadly divided into two categories: the drug-type (marijuana) and the non-drug-type (hemp) [3]. Historical records indicate that *Cannabis sativa* was introduced into Morocco as early as the 7th century, with extensive cultivation established by the 15th century in the Rif Mountains of northern Morocco [4], Over time, the harsh mountain environment, combined with the long-standing expertise of local farmers, fostered the development of unique Moroccan landraces highly adapted to their ecological niche [5] Among these, the “Beldiya” variety, traditionally cultivated for centuries in the Rif and Pre-Rif regions, holds cultural and historical significance. Although it produces comparatively modest amounts of resin, this landrace is well adapted to arid conditions and demonstrates resilience under severe water stress [2], 6], In contrast, hybrid cultivars such as Khardala and Cricutal were introduced into Morocco within the past quarter-century with the aim of increasing resin yield. However, these hybrids display greater sensitivity to drought and require substantially higher water input compared to the traditional Beldiya landrace [7], [8], [9]. For many years, Morocco was among the top countries for illicit cannabis cultivation; according to the United Nations Office on Drugs and Crime (UNODC), it was identified as the leading global producer of cannabis resin in 2020 [10]. Currently, Morocco is transitioning with new government legislation that permits the cultivation of cannabis for cosmetic and medical purposes. Due to previous legal restrictions, Moroccan cannabis varieties have not been extensively studied. The recent changes in legal status have significantly influenced scientific research into the therapeutic potential of *Cannabis sativa* L. Consequently, this study focuses on three drug-type *Cannabis sativa* L. seed varieties cultivated in northern Morocco.

Cannabis seeds, obtained from mature female plants, have historically been undervalued in Morocco, where they were often considered by-products of cultivation. Traditionally, they were either incinerated or used as poultry feed, practices that reflected the limited recognition among local farmers of their nutritional and economic potential [11]. In contrast, at the global level, cannabis seeds are widely appreciated for their remarkable nutritional profile and have long been incorporated into food products, traditional medicine, and animal feed [12]. Phytochemical investigations have revealed that these seeds are rich in diverse bioactive constituents, including fatty acids, lignanamides, terpenoids, proteins, esters, and steroids [13], [14], [15], [16], [17], [18], [19]. Beyond their high nutritional content [3], 14], 18], 20], cannabis seeds have been linked to a variety of health-promoting effects, including blood pressure reduction, restoration of essential fatty acid balance [2], 21], and relief of constipation [22]. *In vitro* studies have demonstrated that extracts from cannabis seeds and their phenolic compounds possess protective capabilities and antiradical [22], [23], [24], [25]. These extracts have also been shown to reduce inflammation and inhibit mediators involved in pain relief [26], [27], [28], [29] and to inhibit cancer cell growth [30], [31], [32]. However, there is a scarcity of studies examining the pharmacological effects of cannabis seed extracts in whole living organisms. Therefore, this study aims to further investigate the phytochemical composition of *Cannabis sativa* L. seeds while evaluating their antioxidant, anti-inflammatory, and anti-nociceptive properties using *in vivo* models.

## 2 Materials and methods

### 2.1 Seed collection

The seeds of three varieties of *Cannabis sativa* L. Cricutal (Cric), Khardala (Khard), and Beldiya (Beld). were collected from the Tafrant region in Taounate, Morocco (34°39′28.4” *N*, 5°05′58.9” W) in September 2021. After collection, the plants were dried at room temperature in a shaded area to preserve their quality. The seeds were then isolated and stored in securely closed plastic bags at room temperature, maintained between 24 °C and 27 °C, until further analysis. The identification of the plant species was conducted by a botanist from the Scientific Institute of Rabat, Morocco. Voucher specimens were archived in the herbarium of the institute with the following identifiers: Bel = RAB 112735, Khard = RAB 112220, Cric = RAB113319.

### 2.2 Extracts preparation

The seeds of three varieties of *Cannabis sativa* L.(Cricutal = Cric, Khardala = Khard, and Beldiya = Beld) were subjected to a washing process with hexane to eliminate any potential THC contamination [4]. Following this, the seeds were ground into a fine powder. A total of 200 g of each powdered sample was macerated in 2000 mL of 70 % ethanol for a duration of 48 h at room temperature, with continuous physical agitation to enhance extraction efficiency. After maceration, the mixture was filtered through Whatman filter paper to separate the solid residues from the liquid extract. The resulting filtrates were concentrated using a rotary evaporator (BUCHI R-205) equipped with a vacuum controller (BUCHI V-805). Subsequently, the concentrated extracts were frozen at −30 °C and then lyophilized to remove any remaining solvent. Ensuring the preservation of bioactive compounds the final products were stored at −4 °C until further analysis. The yield of extraction was as follows (Table 1).

**Table 1:** Yields of the three varieties extracts. expressed as % w/w relative to dry plant material as g extract per g dry weight.

Samples	Yields
Cric	8.10 %
Khard	7.28 %
Beld	8.92 %

### 2.3 Determination of total phenolic content (TPC)

The total phenolic content (TPC) was assessed using the Folin-Ciocalteu reagent, following the method described by Zouhri et al. [33]. Each seed extract (25 μL) was combined with 250 μL of Folin-Ciocalteu reagent (0.2 *N*) and 200 μL of sodium carbonate (Na_2_CO_3_, 3.75 g in 50 mL distilled water). After a 2-h incubation period in darkness at room temperature, the absorbance of the mixture was measured at 760 nm. The results were expressed as milligrams of Gallic acid equivalent per gram of dry weight of the plant (mg GAE/g DWP).

### 2.4 Determination of total flavonoid contents (TFC)

The amounts of flavonoids were estimated spectrophotometrically using a method outlined by Zouhri et al. (2024) [34]. In this procedure, each seed extract (50 μL) was mixed with 150 μL of 2 % aluminum chloride (AlCl3) and 150 μL of 1 % sodium nitrite (NaNO2) in a test tube. The mixture was incubated at room temperature for 1 h to allow for complex formation. After incubation, the optical density was recorded at a wavelength of 510 nm. Quercetin was used to establish a standard curve, and the flavonoid concentration was quantified as milligrams of Quercetin equivalent per gram of dry weight of the plant (mg EQ/g DWP).

### 2.5 High-Performance Liquid Chromatography with Diode Array Detection (HPLC-DAD)

Extracts and standards were prepared at a concentration of 30 mg/mL and filtered through 0.4-μm microfilters to remove any particulate residues prior to injection. The separation and identification of phenolic compounds were conducted using a High-Performance Liquid Chromatography with Diode Array Detection (HPLC-DAD) system (Knaur Platinblue), following the methodology outlined by El-mernissi et al. (2021) with some modifications [35]. Specifically, 20 μL of the seed extract solution was injected into a Kinetex C18 reversed-phase column (250 × 4.6 mm, 2.6 µm particles). The mobile phase consisted of two solvents: acidified water (0.1 % acetic acid), designated as solvent A, and methanol, designated as solvent B. The gradient program was structured as follows: from 0 to 3 min, there was a linear gradient from 5 % to 25 % B; from 3 to 6 min, the composition remained at 25 % B; from 6 to 9 min, it increased from 25 % to 37 % B; from 9 to 13 min, it was held at 37 % B; from 13 to 18 min, the gradient shifted from 37 % to 54 % B; from 18 to 22 min, it was maintained at 54 % B; from 22 to 26 min, it increased from 54 % to 95 % B; from 26 to 29 min, it remained at 95 % B; then from 35 to 35.45 min, it returned to initial conditions at 5 % B; and finally, from 35.45 to 45 min, it stayed at 5 % B. The injection rate was set at 1 mL/min, and the column temperature was maintained at 30 °C. UV–Vis spectral measurements were obtained in the range of 200–400 nm, with chromatographic profiles recorded specifically at 280 nm. The identification of compounds was achieved by comparing the retention times of each peak with those of corresponding standards, ensuring accurate characterization of the phenolic compounds present in the extracts [36].

### 2.6 Antioxidant activity

Four complementary techniques were utilized to assess the antioxidant potential of cannabis seed extracts.

#### 2.6.1 Free radical scavenging (DPPH)

The assessment of 1,1-diphenyl-2-picrylhydrazil (DPPH) free radical scavenging activity of seed samples was monitored using the procedure outlined by Zouhri et al. [37]. The formula employed for calculating the scavenging capacity of the DPPH radical was as follows:

$$\%\;I\text{ DPPH}=\frac{\left(\text{Control}\;\text{absorbance}-\text{samples}\;\text{absorbance}\right)}{\text{Control}\;\text{absorbance}}X\;100$$

The IC_50_ values were determined graphically using linear regression analysis, with Butyl hydroxytoluene (BHT) serving as the positive control.

#### 2.6.2 ABTS radical scavenging assay

The antiradical capability of seed extracts to scavenge 2,2′-azino-bis-(3-ethylbenzothiazoline-6-sulfonic acid (ABTS) radical was assessed using the methodology of EL-Mernissi et al. [38]. After 6 min of incubation, the optical density was measured at 734 nm, and the antioxidant capacity was estimated using the following equation:


$$\%\text{ Inhibition}=\frac{\left(\text{negative}\;\text{Control}\;\text{absorbance}-\text{samples}\;\text{absorbance}\right)\;}{\text{negative}\;\text{Control}\;\text{absorbance}}X100$$

#### 2.6.3 Reducing power

The reducing power assay was conducted following the method outlined by Miguel et al. [39]. The absorbance was determined at 700 nm, The EC50 value was determined graphically and compared with the positive standard (ascorbic acid).

#### 2.6.4 Total antioxidant capacity (TAC)

As previously by Prieto et al., [40] the TAC was assessed. The resulting values were presented as milligrams of ascorbic acid equivalent per gram of dry weight of extract (mg AAE/g DWE).

### 2.7 In vivo anti-nociceptive and Anti-inflammatory activities

#### 2.7.1 Experimental animals

Males Rats weighing between 150 and 200 g were sourced from the animal facility at the Faculty of Sciences Moulay Ismail in Meknes, Morocco. The rats were maintained under standard environmental conditions, with a temperature of 25 ± 1 °C, humidity levels between 55 ± 5 %, and a 12-h light/dark cycle. They had *ad libitum* access to both food and water throughout the study.

**Ethical approval:** The research related to animal use has been complied with all the relevant national regulations and institutional policies for the care and use of animals, and has been approved by the Institutional Ethics Committee for the Care and Use of Laboratory Animals at the Faculty of Sciences, Rabat, within Mohammed V University, Morocco (reference number 86/609/EC20).

#### 2.7.2 Anti-nociceptive activity

The anti-nociceptive activity of the three *Cannabis sativa L.* seed extracts was evaluated using a combination of methods, including one chemical method (the acetic acid-induced writhing test) as described by Koster et al. [41] and two thermal methods: the tail-flick test and the plantar test [42].

##### 2.7.2.1 Tail flick assay (central anti-nociceptive activity)

The tail-flick test was conducted using a analgesymeter (ANALGESYMETER LE 7106, Panlab),equipped with an electrical timer, as described by Sood et al. [43]. The instrument’s heater was maintained at a temperature of 35 ± 0.5 °C, and the final 2 cm of the rats’ tails were uniformly positioned under the heater at the same distance for all subjects. The time interval between the onset of stimulation and the rapid withdrawal of the tail was recorded as tail-flick latency. The experimental groups of rats were pretreated as follows: Group 1 (G1) received 1 mL/100 g of distilled water, Group 2 (G2) received 150 mg/kg of aspirin, Group 3 (G3) received 300 mg/kg of Cric, Group 4 (G4) received 300 mg/kg of Khard, and Group 5 (G5) received 300 mg/kg of Beld. Tail-flick latency was assessed immediately before oral administration and at intervals of 30, 60, 90, 120, 150, and 180 min thereafter.

##### 2.7.2.2 Plantar test (central anti-nociceptive activity)

The test was carried out using the instrument Ugo Basile 37,370 following the method previously reported by Hargreaves [44], 45]. Briefly, rats were kept in Plexiglas boxes with sizes (L = 18 cm, l = 29 cm, h = 12.5 cm). The experimental groups of rats received their extract and drug by gavage as (G1:1 ml/100 g distilled water, G2: 150 mg/kg aspirin, G3:300 mg/kg of Cric, G4: 300 mg/kg of khard, G5: 300 mg/kg of Beld). The movable radiant heater was maintained at 25 ± 0.1 °C, and radiant heat stimulation was applied to the plantar surface of the paw. The heater turned off automatically when the animal lifted its paw. Movement and lapping of the paw were judged to be signs of nociceptive activity. The time between the beginning and the end of the stimulation was recorded as the withdrawal latency and twenty seconds was considered the cut-off. The latency of the paw withdrawal response was measured automatically before and at 30, 60, 90 and 150 min.

##### 2.7.2.3 Acetic acid induced writhing test (peripheral anti-nociceptive activity)

The acetic acid-induced writhing test was conducted as previously described by Koster et al. [41], with some modifications [37]. The animals were administered oral treatments as follows: Group 1 (G1) received 1 mL/100 g of distilled water, Group 2 (G2) received 150 mg/kg of aspirin, Group 3 (G3) received 300 mg/kg of Cric, Group 4 (G4) received 300 mg/kg of Khard, and Group 5 (G5) received 300 mg/kg of Beld. After a thirty-minute period, an intraperitoneal injection of acetic acid solution (0.6 %, 3.75 mL/kg) was administered to induce writhing.

Following the injection, each rat was placed in an individual clear plastic box for 20 min. The number of writhes observed during this period was recorded, and the percentage of inhibition was calculated using the following equation :

$$\%\text{ Inhibcition}=1-\frac{\left(\text{number}\;\text{of}\;\text{writhes}\;\text{in}\;\text{treated}\;\text{group}\;\right)}{\left(\text{number}\;\text{of}\;\text{writhes}\;\text{in}\;\text{control}\;\text{group}\right)}\;X\;100$$

**Figure 1:**
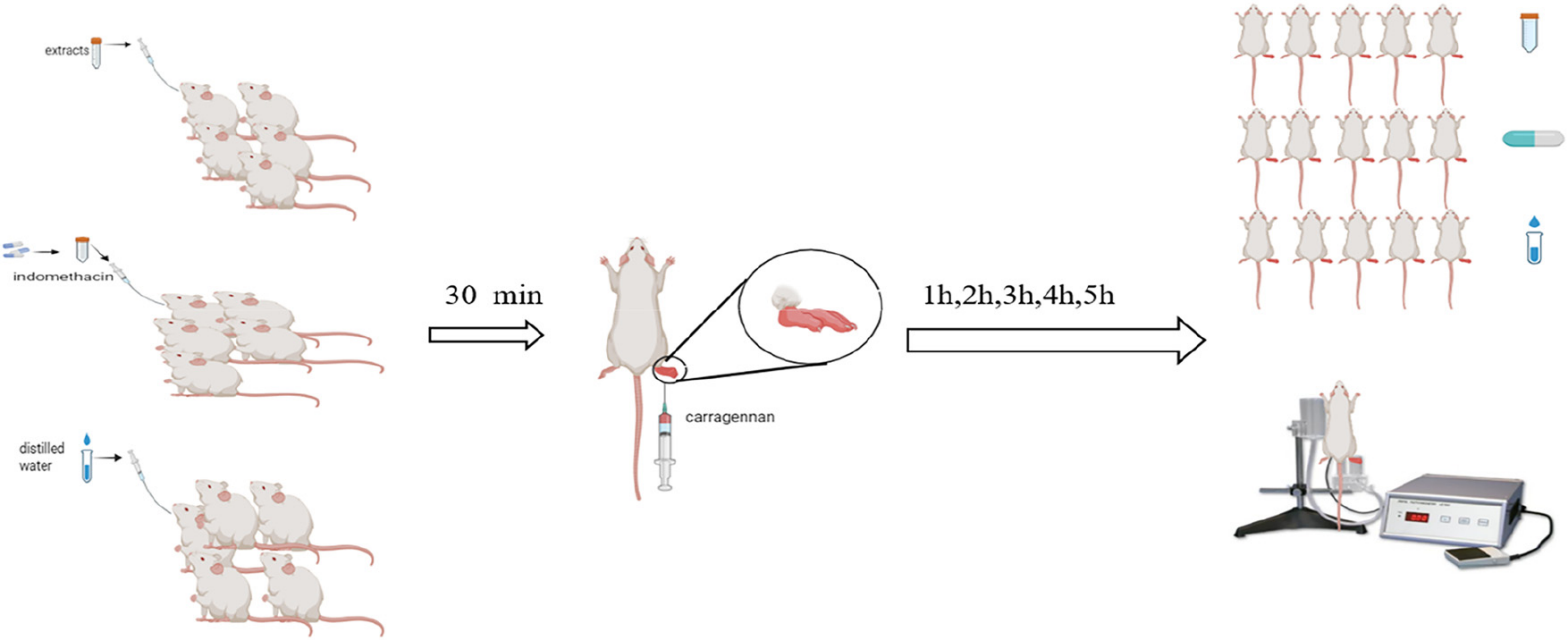
The carrageenan-induced paw edema rat model.

### 2.8 Anti-inflammatory activity

#### 2.8.1 *In vivo* anti-inflammatory activity: carrageenan-induced paw edema assay (Figure 1)

The carrageenan-induced rat paw edema assay was used to assess *in vivo* anti-inflammatory activity following the method described by Winter et al. with slight modification [46], 47] (Figure 1). The edema was produced in the right hind paw by injecting carrageenan (1 % w/v) subcutaneously After 30 min of oral administration of the drug and extracts (G1:1 ml/ss100 g distilled water, G2: 10 mg/kg indomethacine, G3:300 mg/kg of Cric, G4: 300 mg/kg of Khard, G5: 300 mg/kg of Beld), the foot injected size was carried out using plethysmometer (Panlab LE7500) before and 1,2,3,4,5 h after injection in each group. The anti-inflammatory effect was calculated using the following equation:

$$\%\text{ Inhibition}=\frac{\left(\text{Sc}-\text{St}\;\right)}{\text{Sc}\;}\;X\;100$$

With: Sc = size mean of the injected foot in control group

St = size mean of the injected foot in the treated group.

#### 2.8.2 *In vitro* anti-inflammatory activity: bovine serum albumin protein denaturation method

The *in vitro* anti-inflammatory activity was assessed using the Bovine Serum Albumin (BSA) Protein Denaturation Assay, as described by Lekouaghet et al. [48]. For each extract at varying concentrations, 0.5 mL was combined with 0.5 mL of Bovine Serum Albumin (0.2 % w/v) dissolved in Tris buffer (pH 6.8). The reaction tubes were vortexed and then placed in a water bath at 37 °C for 15 min, followed by heating to 75 °C for 5 min. After cooling, the optical density was measured at 660 nm. Control samples were prepared using 0.5 mL of distilled water and 0.5 mL of Bovine Serum Albumin, while blank samples consisted of 0.5 mL of buffer and 0.5 mL of extract, all under identical conditions. Each test was repeated three times, and the percentage inhibition of protein denaturation was calculated using the following formula:

$$\%\text{ inhibition}=\frac{\left[\left(\text{Ac}\right)-\left(\text{As}-\text{Aw}\right)\right]}{\left(\text{Ac}\right)}\times 100$$

Ac = optical density of the control.

As = optical density of the sample.

Aw = optical density of the white.

### 2.9 Docking studies

#### 2.9.1 Protocols for molecular docking

The ligand molecules namely vanillic acid, gallic acid, 3-4-dihydroxybenzoic acid, catechin, syringic acid, P-coumaric acid, quercetin, rosmarinic acid, ursolic acid were identified from HPLC procedure and their structures were drawn and clean up in ChemDraw 20.1.1, [49]. The energy of these structures were minnimized in Chem3D 20.1.1 [50] by applying MM2 force field [50] The pdb format of ligands thus obtained was converted into pdbqt by utilizing AutoDockTools-1.5.7 [51].

#### 2.9.2 Preparation of protein

The ligands were docked against two different proteins to confirm the antioxidant and anti-inflammatory along with nociceptive activity. The pdb format of proteins with PDB ID 3RP8 and 5IKQ were downloaded from https://www.rcsb.org/. The protein structures were prepared using AutoDockTools-1.5.7. Water molecules and other non-protein parts, along with co-crystal ligands, were removed in order to prevent unwanted interactions [52], 53]. Polar hydrogens and Kollman charges were also added to protein structures [53]. Grid parameters were generated [54] and proteins were saved in pdbqt format [55]. The x, y, and z dimensions of the grid box were set to 21.597, 51.877, 17.696 for 5IKQ and 5.923, 26.628, 0.896 for 3RP8, respectively. The size of the x, y, and z coordinates were set to 80, 80, 80.

#### 2.9.3 Molecular docking

The molecular docking method was used to study the nature and strength of interaction between ligand and protein [56]. Docking of ligands with proteins was carried out in Ubuntu 22.04.5 LTS- [57]. The binding energy of ligands docked with respective proteins was obtained. For the validation of docking protocols, the co-crystal ligands of both proteins were redocked [52], 58]. The BIOVIA Discovery Studio 2024 [52] was utilized for visualization of interactions of all compounds between the ligand-protein complexes shown in (Figure S1–Figure S12).

## 3 Statistical analyses

For statistical analysis, GraphPad Prism 9.5 was used. All findings were expressed as mean ± SD. The data were statistically analyzed using a two-way analysis of variance followed by the Tukey test. The Pearson test was used to determine the correlations between biological activities and phenolic substances. The difference was considered statistically significant when p < 0.05.

## 4 Results

### 4.1 Total phenolic & total flavonoid contents

As shown in Table 2, the total phenolic content ranged from 76.89 mg E AG/g to 81.64 mg E AG/g. The highest polyphenol content was observed in the Bled extract, followed by Khard and Cric. In contrast, the highest flavonoid content was found in the Khard extract, followed by Bled and Cric.

**Table 2:** Total polyphenol and total flavonoid content of the three C*annabis Sativa* L seeds hydro-alcoholic extract.

Samples	TFC (mg EQ/g DWP)	TPC (mg E AG/g DWP)
Cric	3.34 ± 0.22^a^	76.87 ± 0.24^a^
Khard	3.56 ± 0.07^a^	81.45 ± 1.37^b^
Beld	3.32 ± 0.12^a^	84.96 ± 2.05^c^

Values in the identical column denoted by the same letter do not exhibit significant differences by Tukey’s multiple range test at p < 0.05. Results are presented as mean ± SD.

### 4.2 Polyphenolic composition

**Figure 2:**
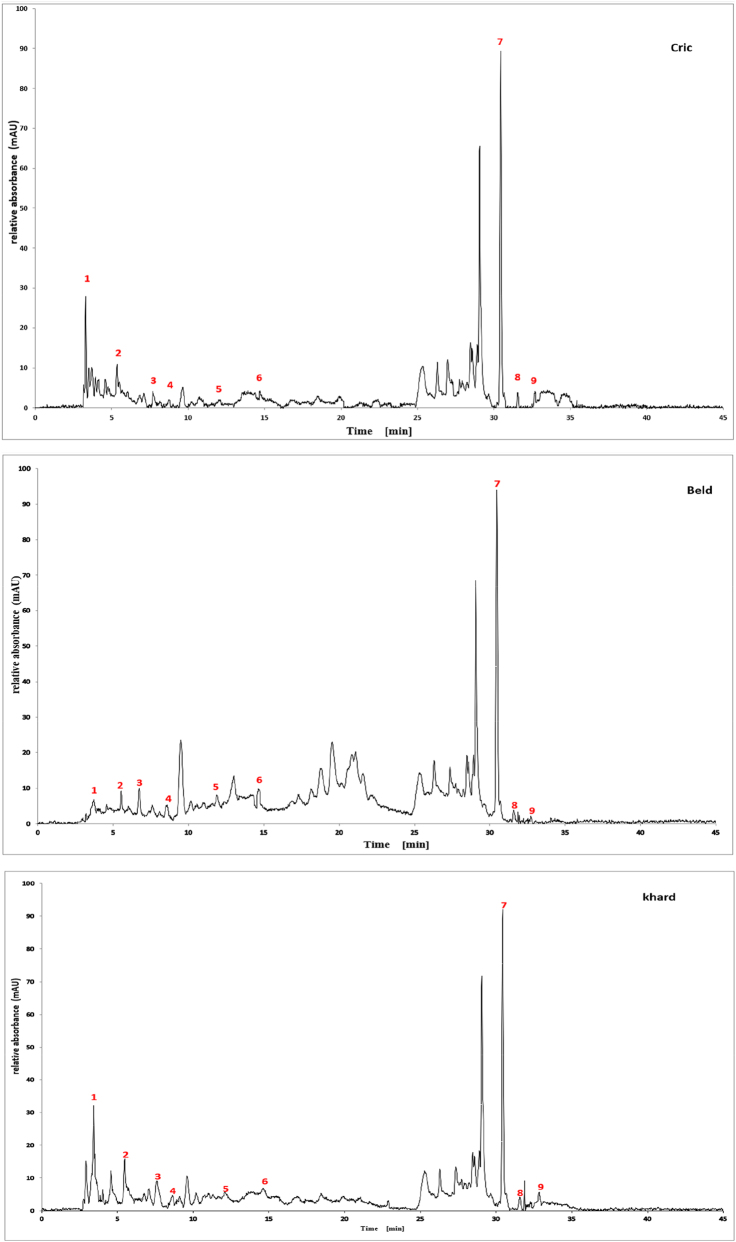
Chromatographic profiles of three *Cannabis Sativa* l seeds (cric, khard, beld) hydro-ethanolic extracts using HPLC-DAD.

The qualitative analysis of the three extracts using High-Performance Liquid Chromatography with Diode Array Detection (HPLC-DAD) is summarized in Table 3. The results revealed the presence of six phenolic acids: gallic acid, 3,4-dihydroxybenzoic acid, syringic acid, *p*-coumaric acid, rosmarinic acid, and vanillic acid; two flavonoids: quercetin and catechin; and one pentacyclic triterpenoid carboxylic acid: ursolic acid. The chromatograms for the three varieties were nearly identical, with slight differences in peak intensity reflecting variations in compound abundance. Notably, quercetin was identified as the predominant phyto-component across all three varieties (Figure 2).

**Table 3:** List of compounds identified in seed extracts using HPLC-DAD.

Pick number	Proposed compounds	Molecular structure	Retention time	Area %		
				Cric	Khard	Beld
1	Vanillic acid	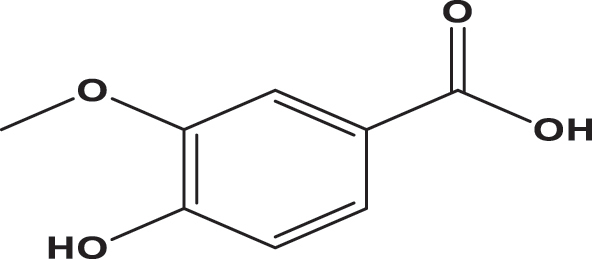	3.35	0.63	3.13	1.93
2	Gallic acid	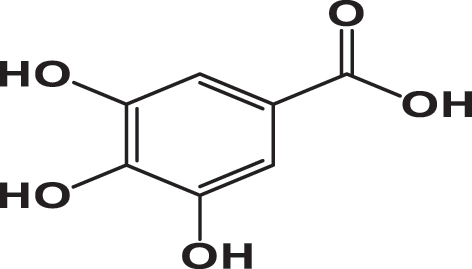	5.45	0.80	1.56	0.98
3	3-4-Dihydroxybenzoic acid	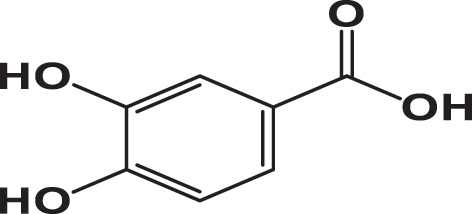	7.68	1.15	0.92	0.31
4	Catechin	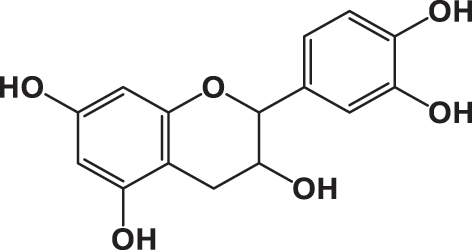	8.50	0.45	0.49	0.12
5	Syringic acid	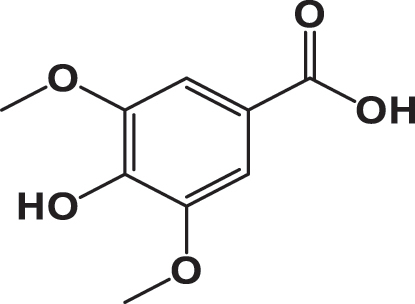	11.98	0.54	0.51	0.17
6	P-comaric acid	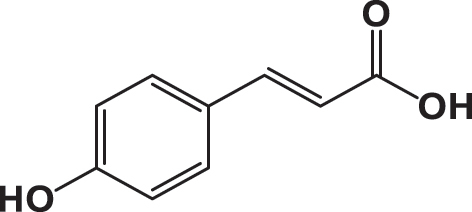	14.68	0.93	0.65	0.33
7	Quercetin	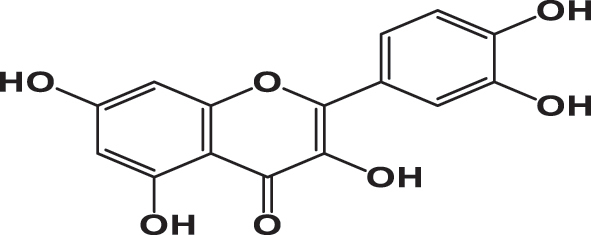	30.38	5.21	5.64	5.81
8	Rosmarinic acid	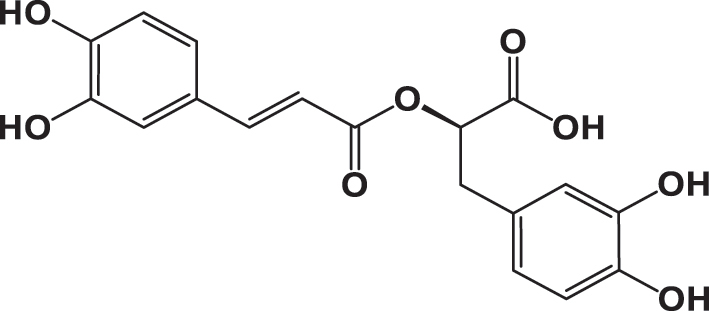	31.6	0.23	0.30	0.31
9	Ursolic acid	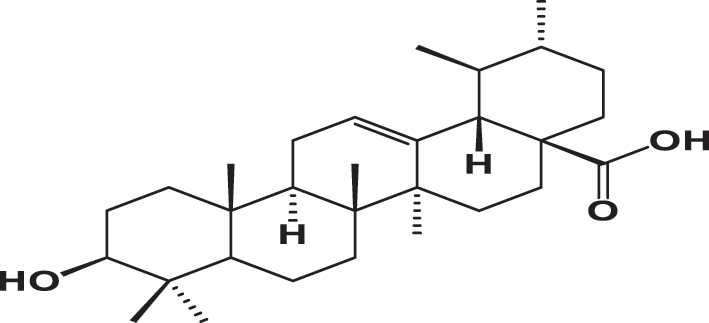	32.75	0.11	0.57	0.28

### 4.3 Antioxidant activity

Table 4 presents the IC50 values for the three varieties, along with standard antioxidant activity for the four methods employed. IC50 values were determined graphically, with lower IC50 values indicating higher antioxidant activity. All varieties demonstrated antioxidant activity, albeit with slight variations among them, which can be attributed to their richness in phenolic components such as phenolic acids, lignans, flavonoids, and stilbenes that react with free radicals either independently or synergistically [59], 60]. The Beld variety exhibited strong antioxidant activity with lower IC50 values, while the Cric variety displayed the weakest activity. Across all five methods tested, the antioxidant activity of the extracts decreased in the following order: standard > Beld > Khard > Cric. This trend aligns with expectations that maximum antioxidant activity corresponds to higher bioactive component content. The results of the antioxidant activity were consistent with those obtained for polyphenol content, as previous studies have highlighted the significance of polyphenols in antioxidant activity [25].

**Table 4:** Antioxidant activities of the three *Cannabis Sativa* L seeds extract.

	DPPH (IC_50_ mg/ml)	ABTS (IC50 mg/ml)	RP (EC50 mg/ml)	TAC (mg AAE/g DWE)
Cric	0.81 ± 01^a^	0.78 ± 004^a^	0.35 ± 005^a^	30.89 ± 0.29^a^
Khard	0.26 ± 008^b^	0.75 ± 006^a^	0.34 ± 003^a^	22.51 ± 0.21^b^
Beld	0.12 ± 007^c^	0.71 ± 001^a^	0.32 ± 004^b^	16.71 ± 0.32^c^
BHT	0.0175 ± 0.002^d^	–	–	–
Acid ascorbic	–	0.053 ± 0.009^b^	0.131 ± 0.025^c^	–

Values in the same column followed by the same letter are not significantly different by Tukey’s multiple range test at p < 0.05, Values are expressed as mean ± SD.

For DPPH free radical scavenging, an antioxidant is considered effective if its IC50 is less than 5 mg/mL. All varieties exhibited a concentration-dependent scavenging potential that increased with rising concentrations. IC50 values ranged from 0.1222 ± 0.073 mg/mL in Beld to 0.81 ± 0.11 mg/mL in Cric. The standard tested (BHT) recorded an excellent inhibitory concentration of 0.0042 mg/mL, surpassing all varieties of extracts.

The results for ABTS were similar across the three varieties. The Beld variety demonstrated the highest ABTS radical cation scavenging activity, with an IC50 of 0.7157 ± 0.0117, followed by Khardala (IC50 = 0.7510 ± 0.062) and Cric (IC50 = 0.7884 ± 0.049).

The reducing power ability of hydro-ethanolic extracts was compared to ascorbic acid as a standard antioxidant. As illustrated in Table 3, reducing levels were higher in the Beld variety compared to Khard and Cric varieties.

The total antioxidant capacity test results indicated that the total antioxidant capacities of the Cric, Khard, and Beld varieties expressed as milligrams of ascorbic acid equivalent per gram of extract (mg AAE/g DE) were 30.89, 22.51, and 16.71, respectively. This finding corroborates results from other tests.

### 4.4 Anti-nociceptive activity

#### 4.4.1 Central anti-nociceptive activity

##### 4.4.1.1 Tail flick test

**Figure 3:**
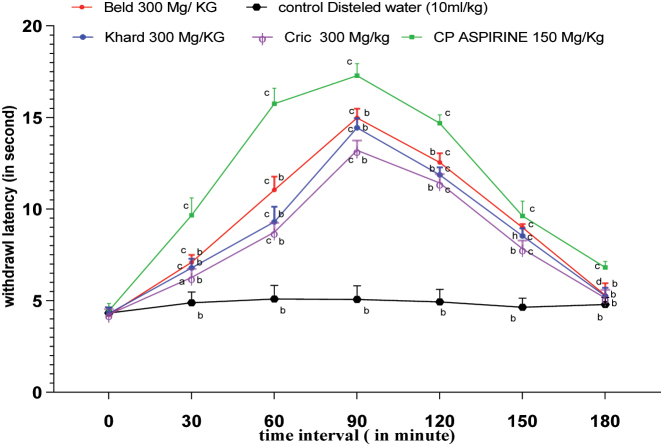
Preventive effect of hydroalcoholic extracts of the three *Cannabis Sativa* L. seeds varieties in the tail flick assay: n = 5 animals/group values are expressed as withdraw latency mean ± SD, ^c^p < 0.0001 significant compared to control group. ^a^p < 0.001 significant compared to control group, ^b^p < 0.0001 significant compared to aspirin group, ^d^p < 0.005 significant compared to aspirin group, ^h^p < 0.01 compared to aspirin group.

The tail flick assay was employed to investigate the spinal responses of rats to heat stimulation [43]. The results for the three different varieties are presented in Figure 3. Tail withdrawal latency increased, peaking at 90 min before beginning to decline, with the Beld variety demonstrating the most significant effect. Aspirin, administered as a standard drug at a dose of 150 mg/kg, along with all varieties’ extracts at a dose of 300 mg/kg, significantly increased (P < 0.0001) withdrawal latency compared to the control group at 60, 90, 120, and 150 min following oral administration. From 30 to 120 min, all three extracts produced a significant increase (P < 0.0001) in tail withdrawal responses compared to the distilled water group.

##### 4.4.1.2 Plantar test

The data for the three varieties of extracts administered orally at a dose of 300 mg/kg are summarized in Table 5. A significant increase in response time was observed for all extracts at 30, 60, 90, 120, and 150 min post-administration (p < 0.0001). The most pronounced effect was noted with the Beld variety (300 mg/kg) at 90 min after gavage.

**Table 5:** Effect of hydro-alcoholic extracts of three *C. sativa L* seeds on planter test in rats.

Samples	Dose	Mean withdrawal latency ± SD in second						
		0 min	30 min	60 min	90 min	120 min	150 min	180 min
Cric	300 mg/kg	2.73 ± 0.63	5.64c ± 0.30	7.04 c ± 0.31	7.86 c ± 0.47	7.62 c ± 0.25	6.06 c ± 0.65	4.08 ± 0.64
Khard	300 mg/kg	3 ± 0.74	5.92c ± 0.70	6.72 c ± 0.41	8.08 c ± 0.58	7.38 c ± 0.43	5.96 c ± 0.50	4.1 ± 0.57
Beld	300 mg/kg	2.28 ± 0,27	6c ± 0.64	7.04 c ± 0.56	9.2 c ± 0.79	7.92 c ± 0,66	6.42 c ± 0.50	4.48 ± 0.72
Aspirin	150 mg/kg	2.64 ± 0.76	5.88 c ± 0.65	7.16 c ± 0.33	9,66 c ± 0.73	8.14 c ± 0.41	6.7c ± 0.64	4.74a ± 0.47
Control group distilled water	1 ml/100 g	2.26 ± 0.59	2.34 ± 0.39	2.34 ± 0.67	2.72 ± 0.67	3.06 ± 0.66	3.52 ± 0.49	3.66 ± 0.48

#### 4.4.2 Peripheral anti-nociceptive activity

##### 4.4.2.1 Acetic acid induced writhing test

**Figure 4:**
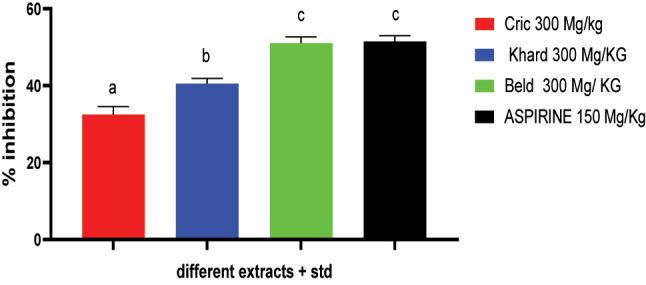
Effects of the hydroalcoholic extract of the three *Cannabis Sativa* L seeds varieties in writhing test in rats, n = 5 animals/group, values are presented as mean ± SD, n = 5 animals/group, different alphabets (a,b,c) represent significant differences (p < 0.005) (one-way ANOVA followed by the Tukey multiple comparison test).

The peripheral anti-nociceptive effects of hydro-ethanolic extracts are summarized in Figure 4. The hydro-ethanolic extracts from the three varieties of *Cannabis sativa L.* seeds demonstrated significant inhibition of pain responses compared to the control group following an intraperitoneal injection of acetic acid. The Cric variety exhibited the weakest effect (32.53 ± 2.09 %), followed by the Khard variety (40.07 ± 1.34 %), while the Beld variety produced the highest inhibition (50.39 ± 1.60 %), which was comparable to the effect of the standard drug (51.19 ± 1.52 %).

### 4.5 Anti-inflammatory activity

#### 4.5.1 *In vivo* anti-inflammatory activity: carrageenan-induced rat paw oedema assay

**Figure 5:**
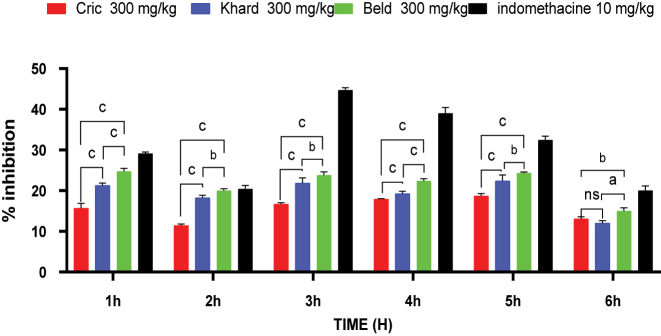
Effect of hydro-alcoholic extracts of the three *C. sativa L* seeds on carrageenan-induced paw edema in rats. c significant at p < 0.0001, a significant at p < 001, b significant at p < 0.05, ns = non-significant.

Figure 5 illustrates the results obtained with the hydro-alcoholic extracts of the three varieties of *Cannabis sativa* L. seeds and the standard drug in the carrageenan-induced edema test. The extracts from all three varieties significantly inhibited paw swelling compared to both the control group and the Indomethacin group from 2 to 6 h after carrageenan injection. The Beld variety exhibited the most pronounced effect.

#### 4.5.2 *In vitro* anti-inflammatory activity: bovine serum albumin protein denaturation method

**Figure 6:**
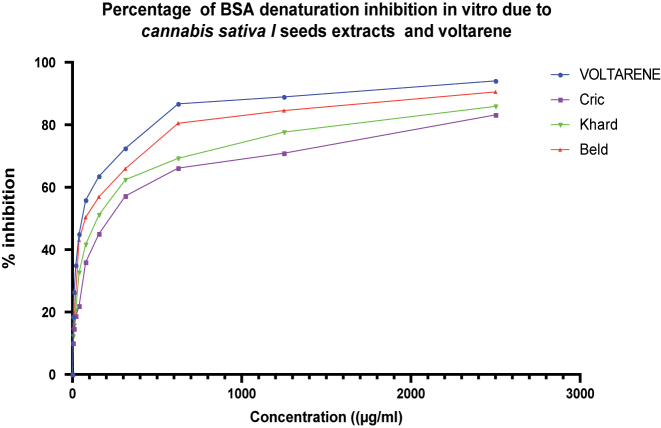
Percentage of BSA denaturation inhibition *in vitro* due *Cannabis Sativa* L seeds extracts and voltarene. Values in the same row followed by the same letter are not significantly different at p < 0.05, values are expressed as mean ± SD.

Protein denaturation is considered a contributing factor to inflammation. The ability of the extract to inhibit protein denaturation was tested as part of the investigation into the mechanism underlying its anti-inflammatory effects. The potential process involved in protein denaturation includes alterations in hydrophobic, hydrogen, disulfide, and electrostatic bonds that maintain the protein’s three-dimensional [61]. The inhibition of bovine serum albumin (BSA) denaturation by various concentrations of the three hydro-alcoholic extracts and the standard drug, Voltaren, is summarized in Figure 6. All extracts effectively prevented BSA protein denaturation.

The current research identified that protein (albumin) denaturation was inhibited in a concentration-dependent manner. The standard drug, Voltaren, at a dose of 2,500 μg/mL exhibited the strongest inhibitory effect against BSA denaturation (94.04 %), followed by the Beld variety (90.53 %), the Khard variety (85.88 %), and the Cric variety (83.16 %). This finding is further supported by comparing their IC50 values presented in Table 6. The presence of polyphenols and flavonoids in *Cannabis sativa* L. seed extracts may account for this beneficial effect [62], 63].

**Table 6:** The 50 % inhibitory concentration (IC_50_) values of BSA denaturation inhibition of three-*Cannabis Sativa* L seeds extracts.

	Cric	Khard	Beld	Voltarene
IC_50_	227.03 ± 4.12^a^	162.38 ± 5.93^b^	89.93 ± 4.33^c^	59.55 ± 4.60^d^

Values in the same row followed by the same letter are not significantly different by Tukey’s multiple range test (p < 0.05).

### 4.6 Correlations of biological activities with phenolic and flavonoid contents

The study aimed to understand the impact of polyphenol and flavonoid content on the biological activities of hydro-ethanolic extracts from *Cannabis sativa* L. seeds. The results revealed a strong negative correlation between the amount of polyphenols and the IC50 values for antioxidant activity measured by DPPH (r^2^ = −0.9678), ABTS (r^2^ = −0.9875), and reducing power (RP) (r^2^ = −0.9647). A similar trend was observed for flavonoids, with negative correlations for antioxidant tests: DPPH (r^2^ = −0.9292), ABTS (r^2^ = −0.9994), and RP (r^2^ = −0.9899) (Table 7). The presence of a phenolic hydroxyl group was identified as a key factor contributing to the high antioxidant activity against DPPH and ABTS radicals [60], 64].

**Table 7:** Pearson correlation coefficients between polyphenol, flavonoids and antioxidant activity (ABTS, RP, DPPH, TAC), BSA denaturation inhibitory activity.

	Polyphenol	Flavonoids	DPPH	ABTS	RP	TAC	BSA inhibitory
Polyphenol	1	0.9923	−0.9678	−0.9875	−0.9647	0.9837	−0.9941
Flavonoids	–	1	−0.9292	−0.9994^a^	−0.9899	0.9984^a^	−0.9999^b^

^a^Correlation is significant at the level p < 0.05. ^b^Correlation is significant at the level p < 0.01.

For bovine serum albumin (BSA) protein denaturation, the IC50 values correlated negatively with flavonoid content (r^2^ = −0.9999, significant at p < 0.01) and polyphenol content (r^2^ = −0.9941). The correlation between total antioxidant capacity (TAC) and flavonoid content was positive (r^2^ = 0.9984) and significant at p < 0.05, while the correlation with polyphenol content was also positive but not statistically significant. The antioxidant activities of phenolic compounds can be attributed to their ability to act as both hydrogen and electron donors simultaneously [65], 66].

### 4.7 *In silico* studies

#### 4.7.1 Interpretation of molecular interactions

By using the molecular docking technique, the ligand-protein binding interactions were predicted and the extent to which interaction occurred. The lower the docking score, the stronger the interaction and greater binding affinity [67]. Table 8 shows the docking score of all compounds obtained from HPLC analysis. The three compounds, quercetin, catechin and ursolic acid, in complex with the 5IKQ (anti-inflammatory and nociceptive) receptor, showed the highest docking scores, −8.9, −8.3 and −8.3 kcal/mol, respectively, as compared to the co-crystallized ligand with docking score −7.3 kcal/mol. Whereas in complex 3RP8 (antioxidant) protein, catechin, quercetin, and rosmarinic acid exhibited higher docking scores with values −7.8, −7.8 and −8.6 kcal/mol, respectively. Hence, the binding energies of (quercetin, catechin, and rosmarinic acid) for antioxidant activity and (quercetin, catechin and ursolic acid) for anti-inflammatory together with nociceptive activity were favourable for efficient docking.

**Table 8:** Docking score of different ligands with the anti-inflammatory and anti-oxidant protein.

Ligand name	Docking score	
	5IKQ	3RP8
Vanillic acid	−5.3	−5.4
Gallic acid	−6.1	−7.0
3-4-Dihydroxybenzoic acid	−5.9	−6.8
Catechin	−8.3	−7.8
Syringic acid	−6.1	−5.6
P-comaric acid	−5.3	−5.7
Quercetin	−8.9	−7.8
Rosmarinic acid	−7.7	−8.6
Ursolic acid	−8.3	−7.1
Co crystallize ligand	−7.3	−12.4

**Figure 7:**
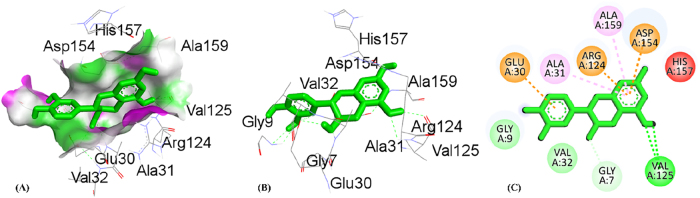
Molecular docking interactions of catechin with the 3RP8 protein, (A) hydrogen bond surface, (B) 3D conformation, and (C) 2D interaction map highlighting key amino acid residues.

**Figure 8:**
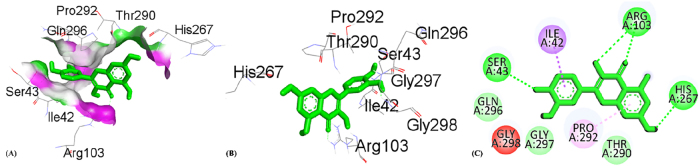
Molecular docking interactions of quercetin with 3RP8 protein. (A) hydrogen bond surface, (B) 3D conformation, and (C) 2D interaction map highlighting key amino acid residues.

**Figure 9:**
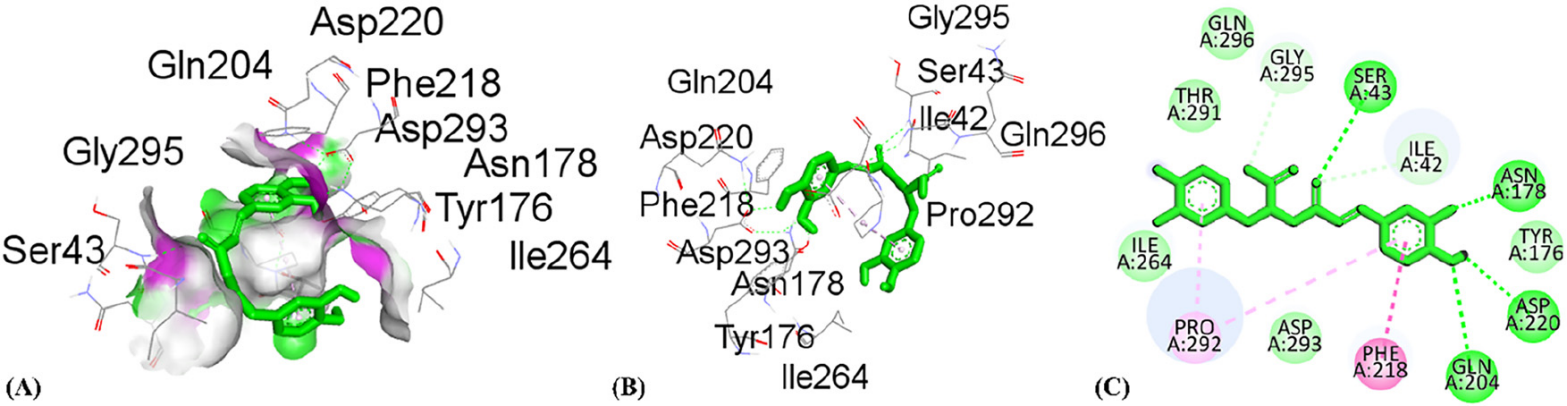
Molecular docking interactions of rosmarinic acid with 3RP8 protein, (A) hydrogen bond surface, (B) 3D conformation, and (C) 2D interaction map highlighting key amino acid residues.

**Figure 10:**
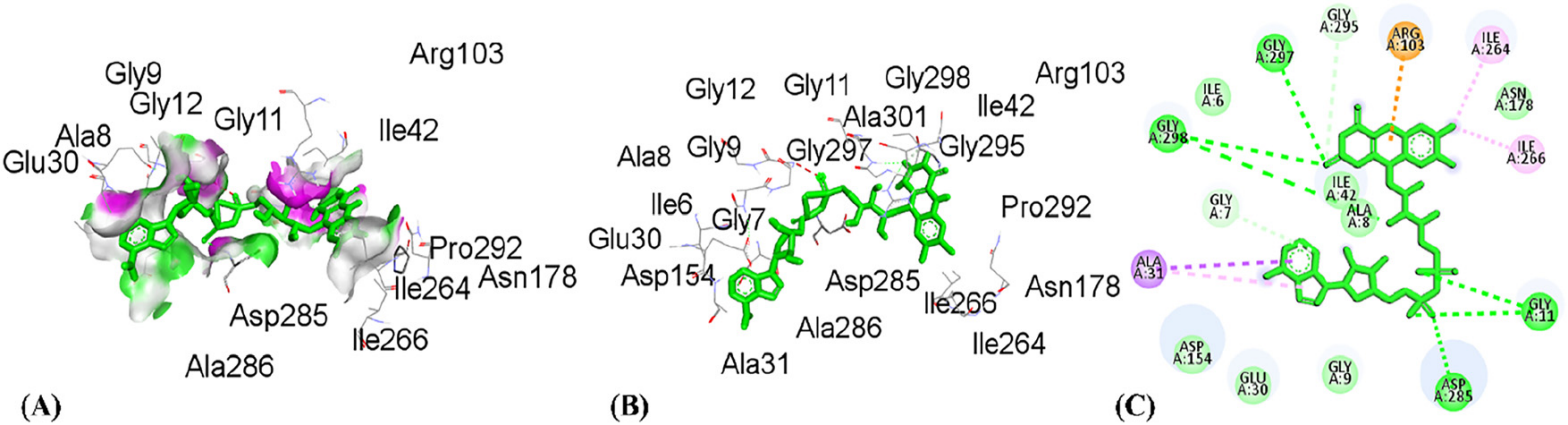
Molecular docking interactions of co-crystalized ligand with 3RP8 protein, (A) hydrogen bond surface, (B) 3D conformation, and (C) 2D interaction map highlighting key amino acid residues.

**Figure 11:**
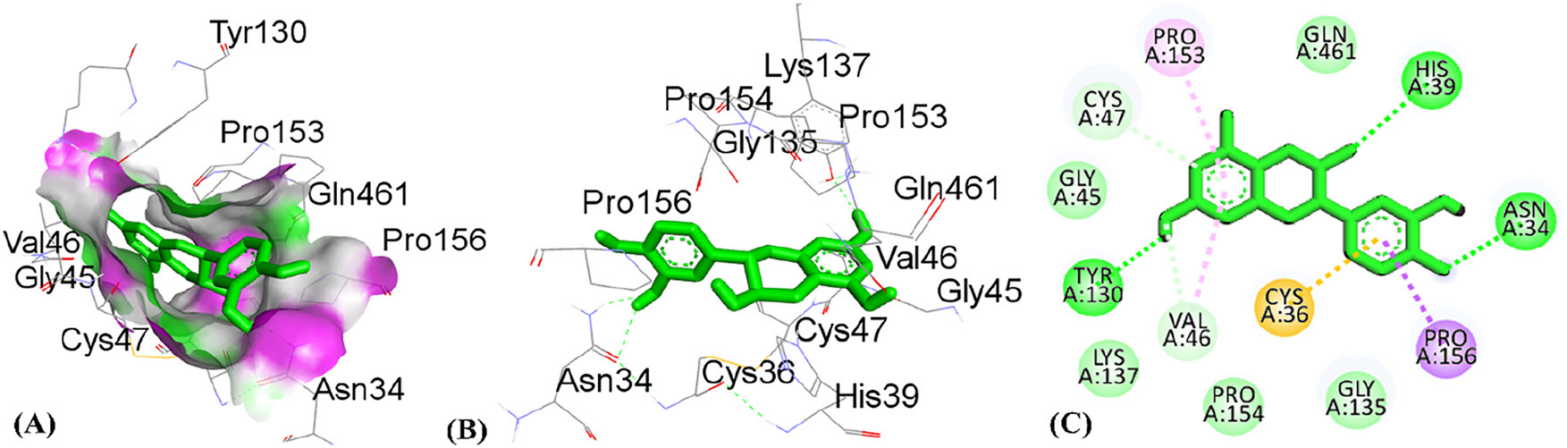
Molecular docking interactions of catechin with 5IKQ protein, (A) hydrogen bond surface, (B) 3D conformation, and (C) 2D interaction map highlighting key amino acid residues.

**Figure 12:**
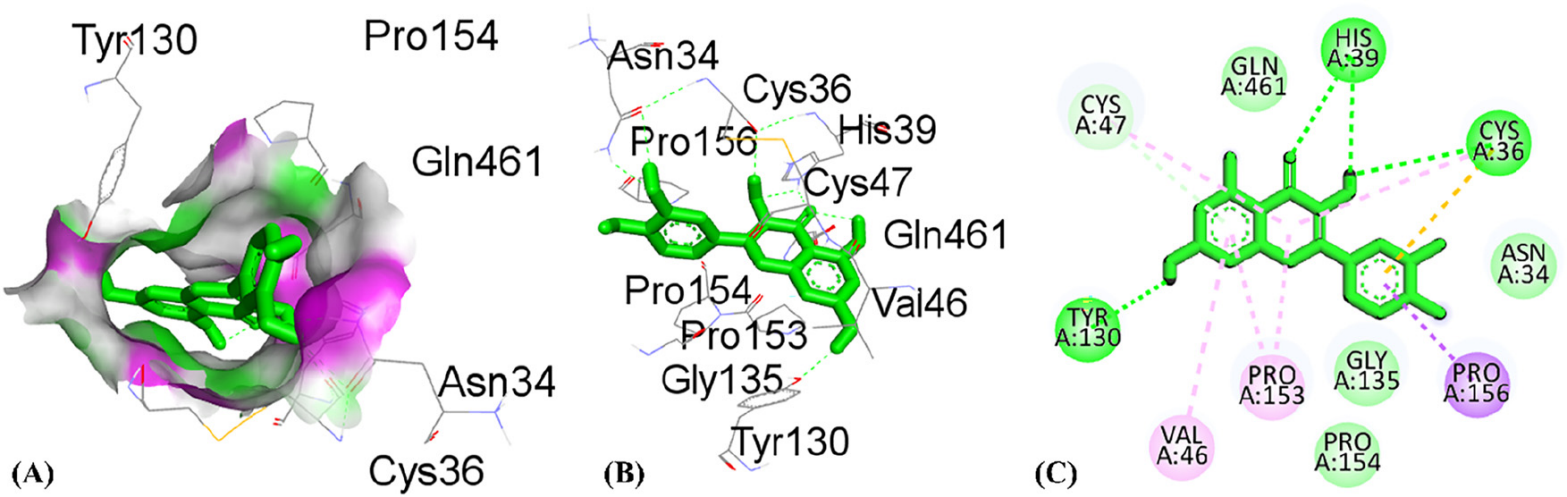
Molecular docking interaction of quercitin with 5IKQ protien, (A) hydrogen bond surface, (B) 3D conformation, and (C) 2D interaction map highlighting key amino acid residues.

**Figure 13:**
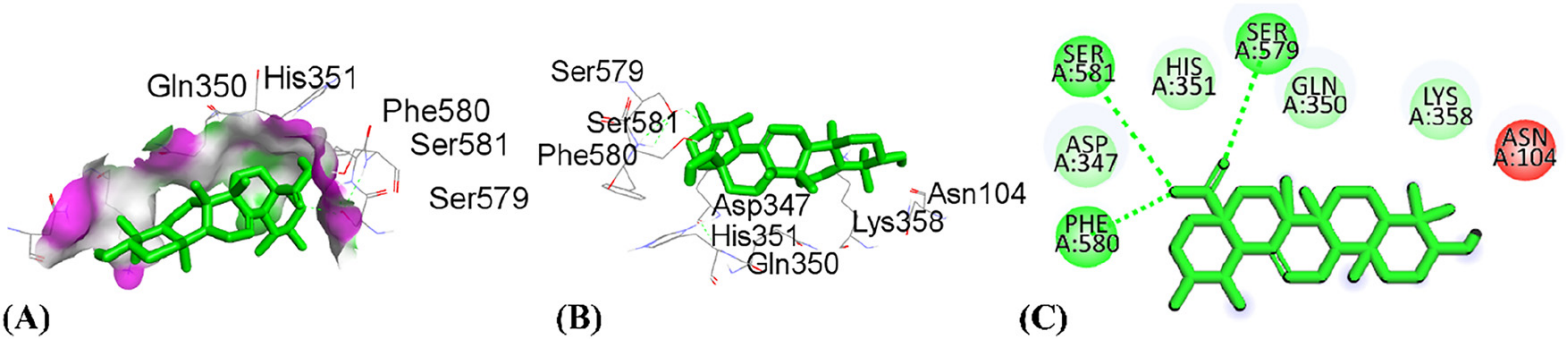
Molecular docking interactions of ursolic acid with 5IKQ protein, (A) hydrogen bond surface, (B) 3D conformation, and (C) 2D interaction map highlighting key amino acid residues.

**Figure 14:**
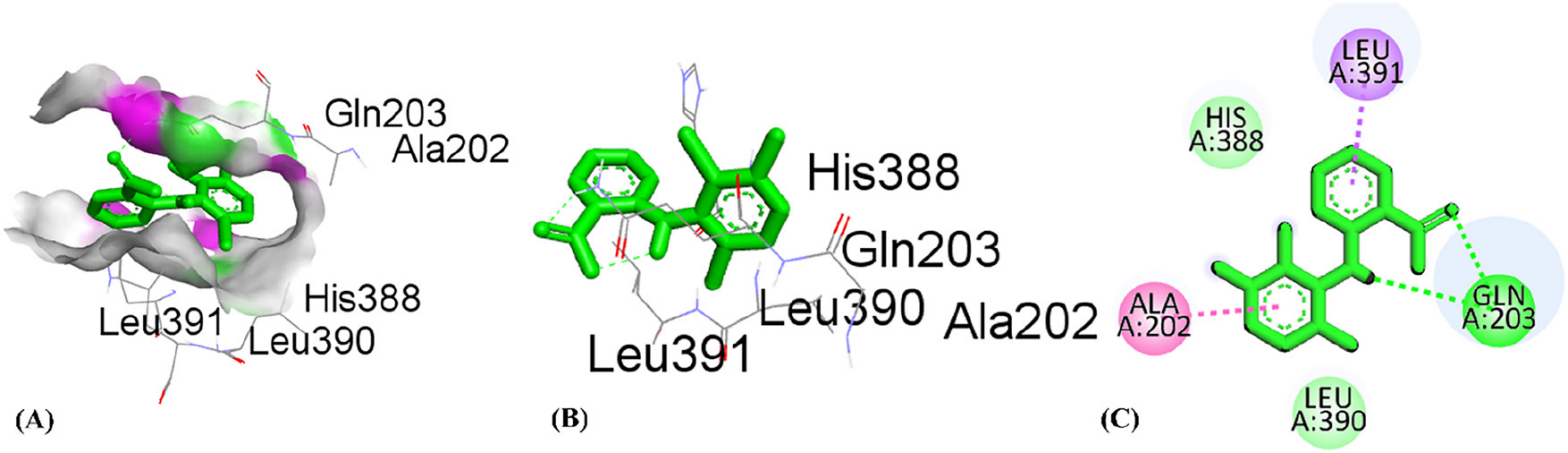
Molecular docking interactions of co-crystalized ligand with 5IKQ protein, (A) hydrogen bond surface, (B) 3D conformation, and (C) 2D interaction map highlighting key amino acid residues.

Table 9 depicted the interacting residues, nature of interaction and distance of top-ranked compounds with the receptors (3RP8 and 5IKQ), while Table S1 depicted the interaction for all compounds extracted from HPLC. The hydrogen bond surface, 2D and 3D interaction of hit and co-crytallized compounds were represented in (Figures 7–14). The catechin showed three types of interaction with the 3RP8 receptor. The residues VAL125, VAL125 and GLY7 represented hydrogen bonds through distances 2.34, 2.02 and 3.51 Å, respectively; Amino acids ALA31, ARG124 and ALA159 showed hydrophobic interactions through the distances of 4.86, 5.28 and 4.39 Å respectively, whereas the electrostatic interactions represented with GLU30 and ASP154 through distance 3.49 and 4.39 Å respectively. The compound quercetin showed hydrogen and hydrophobic interactions with the 3RP8. The quercetin bonded through hydrogen bond to the residues SER43, ARG103, ARG103, and HIS267 with distances 2.42, 2.71, 2.24, and 2.67 Å respectively, while the residues ILE42, PRO292, and PRO292 linked through hydrophobic bonds with distance of 3.62, 5.02 and 4.76 Å respectively. Rosmarinic acid in complex with 3RP8 interacts with the residues SER43, ASN178, GLN204, ASP220, ILE42, and GLY295 by hydrogen bond through distances 2.41, 2.23, 2.88, 2.02, 3.60 and 3.14 Å respectively, while the residues PHE218, PRO292, THR291, PRO292, and PRO292 were connected by hydrophobic interaction through distance 4.02, 5.61, 5.61, 3.78 and 4.40 Å respectively. The co-crystallized ligand (flavin adenine dinucleotide) in 3RP8 showed three types of interactions. First, hydrogen bond interaction displayed with the residues GLY11, GLY155, ALA286, GLY297, GLY298, GLY298, and GLY7 through distance of 2.03, 2.17, 2.14, 2.14, 2.30, 2.35, 2.21, 3.09, and 3.78 Å respectively; second, hydrophobic interactions observed with residues ALA31, ILE264, ILE266, ALA31, PRO292, and PRO292 through distance 3.79, 4.01, 5.25, 4.66, 4.34 and 3.97 Å respectively; whereas the electrostatic interaction exhibited with ARG103 through distance 4.58 Å. The catechin-5IKQ complex showed hydrogen bonding with residues ASN34, HIS39, TYR130, VAL46, CYS47 and PRO156 through distances 2.53, 2.90, 2.06, 3.367, 3.19 and 3.68 Å, respectively; hydrophobic interaction with residues VAL46, CYS47, PRO153 through distance of 5.37, 5.39 and 4.12 Å respectively; and CYS36 displayed other type of interaction with distance 5.37 Å. Quercetin with 5IKQ exhibited hydrogen bond with residues HIS39, HIS39, CYS36, TYR130, and CYS47 through distance of 1.84, 2.49, 2.17, 1.93, 3.31 Å respectively, while the residues PRO156, CYS36, CYS36, CYS47, PRO153, VAL46, and PRO153 displayed hydrophobic interactions through distance 3.71, 5.24, 5.38, 4.88, 4.09, 5.34, and 4.029 Å respectively. Whereas the Ursolic acid bounded with a hydrogen bond to SER579, PHE580, and SER581 residues of 5IKQ through distances 2.33, 2.71 and 2.95 Å, respectively. The co-crystal compound (meclofenamic acid) interacted through hydrogen and hydrophobic interactions with the 5IKQ protein. The residues GLN203, GLN2031 and LEU391 displayed hydrogen interaction with distance 2.39, 2.99 and 2.29 Å respectively, while the hydrophobic interactions observed with residues ALA202, GLN203, ALA202 through distance 3.35, 4.83 and 4.99 Å respectively.

**Table 9:** The interacting amino acid residues along with the type of interaction and distance with docked proteins (3RP8 and 5IKQ).

Protein name	Ligand name	Residues	Type of interaction	Distance (Å)
3RP8	Cathechin	VAL125 VAL125 GLY7 ARG124 GLU30 ASP154 ALA31 ARG124 ALA159	Hydrogen bond Hydrogen bond Hydrogen bond Electrostatic Electrostatic Electrostatic Hydrophobic Hydrophobic Hydrophobic	2.34025 2.02006 3.51178 4.16719 3.4979 4.39754 4.86314 5.28343 4.39453
	Quercetin	SER43 ARG103 ARG103 HIS267 ILE42 PRO292 PRO292	Hydrogen bond Hydrogen bond Hydrogen bond Hydrogen bond Hydrophobic Hydrophobic Hydrophobic	2.41968 2.70656 2.23776 2.67425 3.62366 5.02282 4.75845
	Rosmarinic acid	SER43 ASN178 GLN204 ASP220 ILE42 GLY295 PHE218 THR291, PRO292 PRO292 PRO292	Hydrogen bond Hydrogen bond Hydrogen bond Hydrogen bond Hydrogen bond Hydrogen bond Hydrophobic Hydrophobic Hydrophobic Hydrophobic Hydrophobic	2.40647 2.23576 2.88128 2.02373 3.60182 3.14123 4.02043 5.61317 5.61317 3.78811 4.40407
	Co-crystalized ligand	GLY11 GLY11 GLY11 GLY155 ALA286 GLY297 GLY298 GLY298 GLY7 ARG103 ALA31 ILE264 ILE266 ALA31 PRO292 PRO292	Hydrogen bond Hydrogen bond Hydrogen bond Hydrogen bond Hydrogen bond Hydrogen bond Hydrogen bond Hydrogen bond Hydrogen bond Electrostatic Hydrophobic Hydrophobic Hydrophobic Hydrophobic Hydrophobic Hydrophobic	2.03979 2.16501 2.14165 2.14626 2.3027 2.35133 2.21326 3.09375 3.78274 4.58239 3.79526 4.01153 5.25481 4.65865 4.33637 3.97025
5IKQ	Cathechin	ASN34 HIS39 TYR130 VAL46 CYS47 PRO156 CYS36 VAL46 CYS47 PRO153	Hydrogen bond Hydrogen bond Hydrogen bond Hydrogen bond Hydrogen bond Hydrophobic Other Hydrophobic Hydrophobic Hydrophobic	2.53023 2.90571 2.05782 3.36691 3.196 3.68234 5.36864 5.37353 5.39378 4.11906
	Quercetin	HIS39 HIS39 CYS36 TYR130 CYS47 PRO156 CYS36 CYS36 CYS47 PRO153 VAL46 PRO153	Hydrogen bond Hydrogen bond Hydrogen bond Hydrogen bond Hydrogen bond Hydrophobic Other Hydrophobic Hydrophobic Hydrophobic Hydrophobic Hydrophobic	1.84143 2.48979 2.16904 1.93034 3.31357 3.71103 5.24117 5.3852 4.88241 4.08774 5.33748 4.02854
	Ursolic acid	SER579 PHE580 SER581	Hydrogen bond Hydrogen bond Hydrogen bond	2.33279 2.70553 2.95055
	Co-crystalized ligand	GLN203 GLN2031 LEU391 ALA202 GLN203 ALA202	Hydrogen bond Hydrogen bond Hydrogen bond Hydrophobic Hydrophobic Hydrophobic	2.39033 2.997 2.29919 3.35419 4.82887 4.99407

## 5 Discussion

Phenolic compounds are secondary metabolites found in various plants, known for their ability to neutralize free radicals, modulate the activity of genes involved in metabolism, and consequently protect DNA from damage [68]. Additionally, these compounds can act as signaling molecules that enhance the antioxidant defense system [69], 70]. They exhibit a wide range of pharmacological effects, including cardioprotective, anti-aging, anti-carcinogenic, anti-inflammatory, anti-atherosclerotic, and anti-apoptotic properties. Furthermore, they promote endothelial function and can inhibit processes such as angiogenesis and cell proliferation [62], [71], [72], [73].

In the present study, the levels of phenolic compounds detected were generally lower than those reported in several earlier investigations. For example, Taaifi et al. (2021) quantified phenolic content at 134.57 mg GAE/g DWP [4], while Bhatt et al. (2022) recorded 121 mg GAE/g DWP [74]. Similarly, Haddou et al. (2023) observed values of 130 mg GAE/g DWP in ethanolic extracts, contrasting sharply with only 28 mg GAE/g DWP in aqueous preparations [15]. In comparison, our results were closer to those of Rashid et al. (2020), who reported a total phenolic content of 77.7 mg GAE/g DWP [25], yet still higher than the concentrations documented by Aloo et al. (2023) and Irakli et al. (2019) [75], 76]. Seed morphology also appears to play a role in phenolic distribution: Alonso-Esteban et al. (2022) demonstrated that intact hemp seeds contain higher polyphenol levels than dehulled seeds [32], a finding consistent with Chen et al. (2012) [22], who highlighted the hull as a key polyphenol reservoir. Beyond seeds, Ahmad et al. (2019) reported that the leaves of *Cannabis sativa L.* harbor a richer polyphenolic profile than other plant organs [77].

Flavonoids, a subgroup of phenolic compounds and well-known plant secondary metabolites, contribute significantly to the pigmentation of leaves, fruits, and flowers, while also exerting potent antioxidant activities both *in vivo* and *in vitro* [78], [79], [80], In our samples, flavonoid levels were comparatively modest. Taaifi et al. (2021) documented higher values, ranging between 30 and 50 mg EQ/g DWP [4], whereas Aloo et al. (2023) reported extremely low levels, approximately 1 mg EQ/g DWP [75]. Our findings therefore occupy an intermediate range, suggesting that variations in extraction methods, seed fractionation, and environmental factors may largely account for discrepancies across studies.

The phytochemical profile of our samples is consistent with findings reported in previous studies. For instance, Nigro et al. (2022) detected quercetin and kaempferol derivatives in hempseed extracts, corroborating the presence of flavonoids in our analysis [81]. Similarly, Haddou et al. (2023) characterized 14 distinct phenolic constituents in *Cannabis sativa L.* seeds, with catechin acid dihydrate emerging as the major compound in dichloromethane extracts, whereas naringin was dominant in aqueous and ethanolic fractions. Their work, along with ours, reinforces the critical influence of extraction solvent on the qualitative and quantitative composition of seed phytochemicals [15]. Other investigations have likewise reported variability in dominant phenolics: catechin has frequently been described as the principal phenolic in hemp seed flour [19], 82].Moreover, Benkirane et al. (2022) identified hydroxycinnamic acid amides and lignanamides, particularly cannabisins, as major constituents [83], while Babiker et al. (2021) reported gallic acid, syringic acid, catechin, and 1,2-dihydroxybenzoic acid as key metabolites which aligns with our findings [84].

The phytocompounds identified in the cannabis seeds are known to possess diverse biological activities, including antioxidant, anti-inflammatory, and anti-nociceptive properties, which were explored in this study. For instance, quercetin, the major component found in all varieties, is recognized as a potent free radical scavenger and a long-acting anti-inflammatory agent among flavonoids [85]. Its efficacy is attributed to its chemical structure, which includes four hydroxyl groups on the benzo-dihydropyran ring of the polyphenol [86]. Similarly, several studies have reported that gallic acid exhibits strong anti-inflammatory, anti-tumoral, and anti-nociceptive activities [86]. The significant presence of *p*-coumaric acid, ursolic acid, and syringic acid in cannabis seed extracts suggests a strong potential for exerting potent anti-nociceptive and anti-inflammatory effects [87], [88], [89], [90]. Furthermore, the presence of compounds such as vanillic acid, rosmarinic acid, and catechin within these extracts indicates their potential roles in the observed pharmacological activities [91], [92], [93].

It is widely acknowledged that reactive oxygen species (ROS) are produced during oxidative stress. These radicals can be highly toxic to various molecules, leading to cellular dysfunction and, in some cases, cell death [94]. Antioxidants are compounds that directly neutralize free radicals and facilitate the removal of reactive species from cells. The extensive use of traditional medicine underscores the importance of plants as a rich source of natural antioxidants, which may guide the development of innovative pharmaceuticals [95]. Several investigations have highlighted the antioxidant potential of *Cannabis sativa* L. seeds, though reported outcomes vary considerably across studies. Alonso et al. evaluated hydro-ethanolic extracts from seven cultivars and found relatively modest activity, with reducing power IC_50_ values between 2.5 and 5.3 mg/mL and weak DPPH radical scavenging activity, yielding IC_50_ values in the range of 2.5–9.2 mg/mL [22]. These values are noticeably higher than those recorded in the present work. In contrast, Bhatt et al. demonstrated remarkable DPPH scavenging capacity, reporting an IC_50_ of approximately 50 μg/mL [74], suggesting that cultivar differences and extraction approaches may significantly influence outcomes. Beyond crude seed extracts, cannabis seed protein hydrolysate fractions have been reported to display strong antioxidant properties, further supporting the role of seed-derived bioactive peptides in oxidative stress modulation [96], [97], [98]. Additionally, investigations into hemp seed oil by Smeriglio et al. revealed even greater antioxidant activity than that observed in our study, underscoring the contribution of lipid-soluble phytochemicals to the antioxidant potential of cannabis seeds [99].

Pain can be categorized into several types: nociceptive pain, which is triggered by harmful physical stimuli; inflammatory pain, arising from the immune system’s response to tissue injury; and neuropathic pain, which results from damage to the nervous system due to physical injury or disease affecting the somatosensory system [100]. Analgesics are medications designed to alleviate pain selectively without significantly affecting consciousness, acting on either the peripheral or central nervous system [101]. However, analgesic drugs such as non-steroidal anti-inflammatory drugs (NSAIDs) and opioids are associated with severe adverse effects [102]. Therefore, it is crucial to conduct research aimed at identifying alternative treatments for pain management. For centuries, medicinal herbs have been utilized for therapeutic purposes. Various compounds isolated from these plants such as alkaloids, flavonoids, steroids, and tannins have demonstrated notable anti-nociceptive properties [103], 104]. In this context, the hydro-ethanolic extracts of the three varieties were effective in the tail flick test, Writhing test, and plantar test. In this study, the anti-nociceptive activity of *Cannabis sativa* L. seed extracts was evaluated through established models that assess both central and peripheral pain responses, The acetic acid induced writhing test, for instance, stimulates the release of several inflammatory mediators including cytokines, histamine, bradykinin, and serotonin [105], and increases the levels of prostanoids (prostaglandin E2 (PGE_2_) and prostaglandin F2α (PGF_2_α)) as well as lipoxygenase products in the peritoneal cavity [106], The observed anti-nociceptive effect of the extracts may therefore be linked to phytoconstituents capable of inhibiting the prostaglandin pathway and attenuating the release of pro-inflammatory intermediates, In addition, flavonoids such as catechin, naringin, and quercetin detected in cannabis seed extracts [15], 82], 107], 108] have been shown to modulate central nociceptive pathways. These compounds act, at least in part, through alpha-2 adrenergic [109], 110] and opioid receptors [111], thereby contributing to the central pain-relieving effects. Taken together, our findings indicate that *Cannabis sativa* L. seed extracts exert analgesic activity via both peripheral and central mechanisms of action, supporting their potential as sources of bioactive compounds for pain management.

Inflammation is a multifaceted biological response triggered by infection, injury, or tissue damage and is frequently implicated in chronic conditions such as cancer, diabetes, and neurodegenerative disorders [112]. To investigate anti-inflammatory activity, the carrageenan-induced paw edema model in rats is one of the most widely employed experimental systems, with paw volume serving as a reliable indicator of treatment efficacy [113], 114].Carrageenan acts as a strong pro-inflammatory agent, provoking edema through the upregulation of cyclooxygenase (COX) messenger RNA [115], and the subsequent release of mediators such as leukotrienes, tumor necrosis factor-α (TNF-α), histamine, and prostaglandins [114]. This inflammatory cascade is further amplified by the generation of reactive oxygen species (ROS), which contribute to tissue damage and the progression of inflammation [116].The notable ability of the three cannabis seed extracts to scavenge free radicals suggests that this may contribute to the reduction of inflammation observed in rats with carrageenan-induced paw edema [117]. Furthermore, it has been reported that lignin amide rich fractions extracted from *Cannabis sativa* L. seeds demonstrate anti-inflammatory potential by suppressing Toll-like receptor 4-mediated NF-κB signaling pathways [118]. Rea Martinez and colleagues documented the anti-inflammatory effects of polyphenol-rich fractions from hemp seeds by reducing the expression and secretion of TNF-α and IL-6 genes [119]. Prior studies have also suggested that phenolic acids, flavonoids, and fatty acids present in *Cannabis sativa* L. seed extracts may interact directly with the prostaglandin system [120], [121], [122]. Additionally, these compounds may inhibit xanthine oxidoreductase activity, block nuclear factor κB (NF-κB) pathways, and inhibit cyclooxygenase (COX) enzymes [63], 120], [123], [124], [125], [126] The observed effects may result from a synergistic interaction among multiple phyto-constituents rather than from a single compound [127].

## 6 Conclusions

The current research concluded that *Cannabis Sativa* L. seeds are rich in polyphenols. On the other hand, hydro-ethanolic extract of the three varieties have remarkable antioxidant, anti-nociceptive and anti-inflammatory activities examined by various tests along with a silico study; the beldiya variety exhibits the highest levels of total phenol and flavonoid contents as well as the best antioxidant, anti-inflammatory properties and anti-nociceptive effect.

According to the findings of this study, the *Cannabis Sativa* L seeds are suggested as a dietary additive, without omission of their phytochemical component relevance in pain-relieving and inflammatory therapy.

## Supplementary Material

Supplementary Material
