# Supplementary material for: A comparative study on phytochemical analysis and biological properties of three varieties of cannabis sativa L. seeds
Source: Open Life Sci. 2026 Feb 6;21(1):20251211. doi: 10.1515/biol-2025-1211 (PMC12917594; doi:10.1515/biol-2025-1211)
Supplement: Supplementary file 1 — Supplementary Material [file j_biol-2025-1211_suppl_001.docx]

**A comparative study on Ionomic, phytochemical analysis and biological properties of three verities of *cannabis sativa* L.**

**Supplementary material**

Table S 1 : The interacting amino acid residues along with the type of interaction and distance for HPLC derived compounds with proteins (3RP8 and 5IKQ)

| **Ligand name** | **residue** | **Type of interaction** | **Distance (Å)** |
| --- | --- | --- | --- |
| **3RP8** | | | |
| Ascorbic acid | ARG171  HIS267  GLU265  THR290  THR290  HIS267 | Hydrogen Bond  Hydrogen Bond  Hydrogen Bond  Hydrogen Bond  Hydrogen Bond  Hydrogen Bond | 2.591  2.09437  2.39827  2.18429  2.18129  2.03628 |
| Gallic acid | TYR176  GLN204  GLN204  ASP293  ASP293  ASP220  PHE218  PRO292 | Hydrogen Bond  Hydrogen bond  Hydrogen bond  Hydrogen Bond  Hydrogen Bond  Hydrogen Bond  Hydrophobic  Hydrophobic | 2.03002  2.52  2.73932  2.91523  2.20364  2.27942  4.06161  4.48575 |
| 3-4-Dihydroxybenzoic acid | TYR176  GLN204  GLN204  ASP220  PHE218  PRO292 | Hydrogen Bond  Hydrogen Bond  Hydrogen Bond  Hydrogen Bond  Hydrophobic  Hydrophobic | 2.07727  2.68489  2.98418  2.66094  3.92736  4.36723 |
| Syringic acid | ARG103  ARG103  GLN107  ASP285  ASP285  ASP285  ALA153  ASP285  ILE10  ALA301 | Hydrogen Bond  Hydrogen Bond  Hydrogen Bond  Hydrogen Bond  Hydrogen Bond  Hydrogen Bond  Hydrogen Bond  Electrostatic  Hydrophobic  Hydrophobic | 2.39549  2.14357  2.73649  2.36034  2.76866  3.62789  3.49851  4.64961  3.87967  5.17476 |
| p-comaric acid | SER43 | Hydrogen Bond | 1.93298 |
| Ursolic acid | ARG263  GLU265 | Hydrogen Bond  Hydrogen Bond | 2.74111  2.67074 |
| **5IKQ** | | | |
| Ascorbic acid | TRP387 | Hydrogen Bond | 2.16298 |
| Gallic acid | THR206  ALA199  HIS207  ALA202 | Hydrogen Bond  Hydrogen Bond  Hydrogen Bond  Hydrophobic | 2.74572  1.90453  3.55194  5.18906 |
| 3-4-Dihydroxybenzoic acid | ALA202, GLN203  ALA202 | Hydrophobic  Hydrophobic | 4.54092  4.68805 |
| Syringic acid | ALA202  ALA199  ALA202, GLN203  ALA199  LEU390  LEU391  HIS207  HIS388 | Hydrogen Bond  Hydrogen Bond  Hydrophobic  Hydrophobic  Hydrophobic  Hydrophobic  Hydrophobic  Hydrophobic | 2.14583  3.51755  5.20627  3.66197  4.22859  4.5159  5.44289  4.55816 |
| P-comaric acid | SER579  GLN350  HIS351  GLN350, HIS351 | Hydrogen Bond  Hydrogen Bond  Hydrophobic  Hydrophobic | 2.32702  2.32806  3.77248  5.1569 |
| Rosmarinic acid | GLN203  HIS207  HIS388  TRP387  GLN203  HIS386  LEU391  VAL444  ALA202 | Hydrogen Bond  Hydrogen Bond  Hydrogen Bond  Hydrogen Bond  Hydrogen Bond  Hydrogen Bond  Hydrophobic  Hydrophobic  Hydrophobic | 3.0352  3.07968  2.68715  2.54561  2.36075  2.35655  4.54227  5.45368  5.49048 |


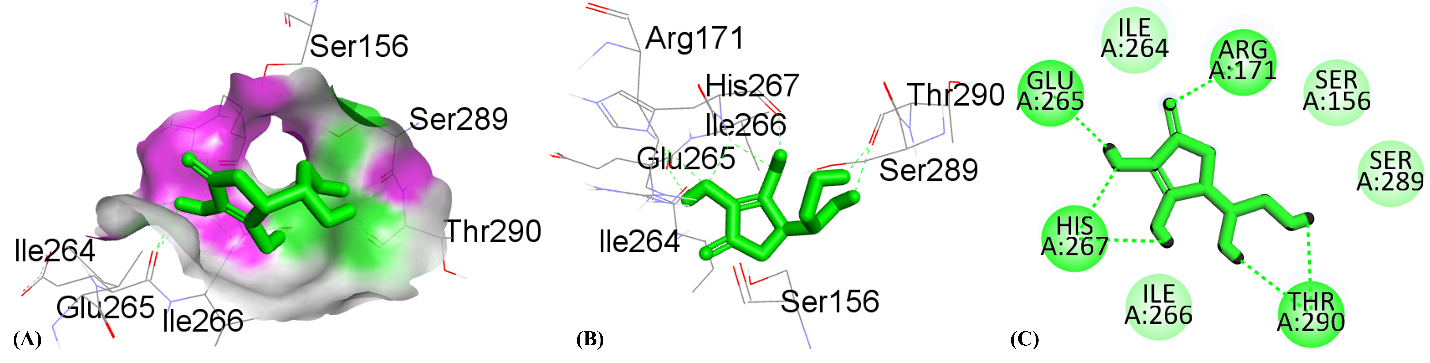


Figure S 1 : (A) hydrogen surface (B) 3D and (C) 2D interaction of ascorbic acid in complex with 3RP8


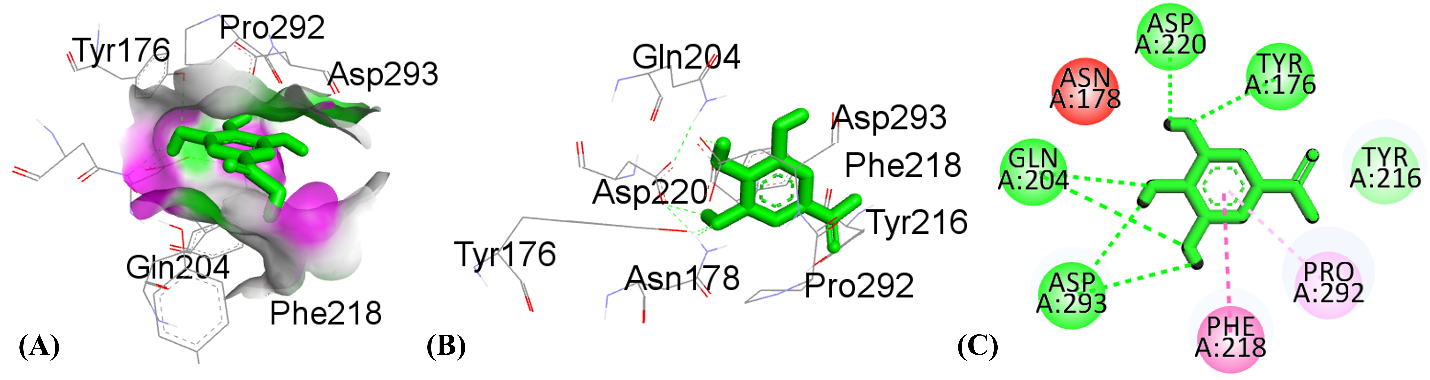


Figure S 2 : (A) hydrogen surface (B) 3D and (C) 2D interaction of gallic acid in complex with 3RP8


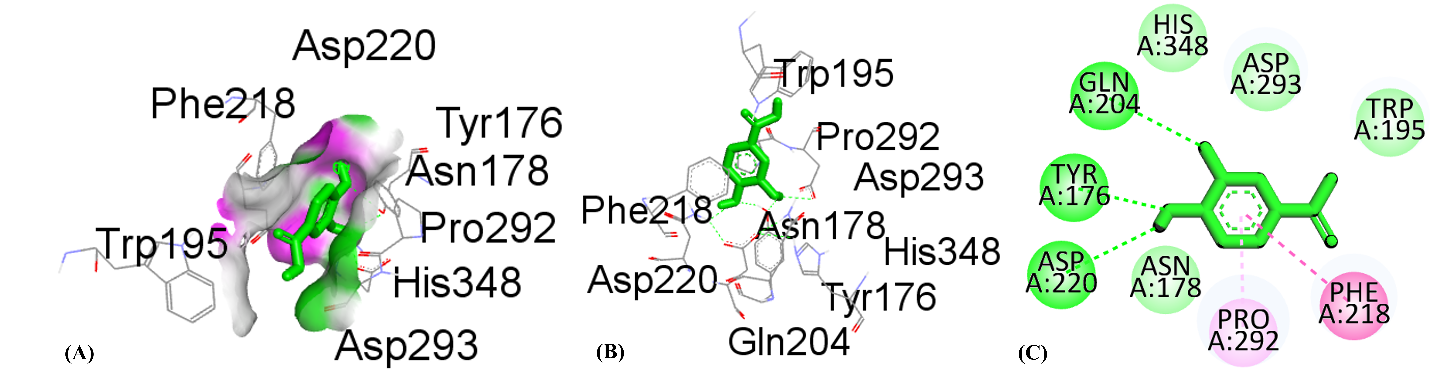


Figure S 3 : (A) hydrogen surface (B) 3D and (C) 2D interaction of 3-4-Dihydroxybenzoic acid in complex with 3RP8


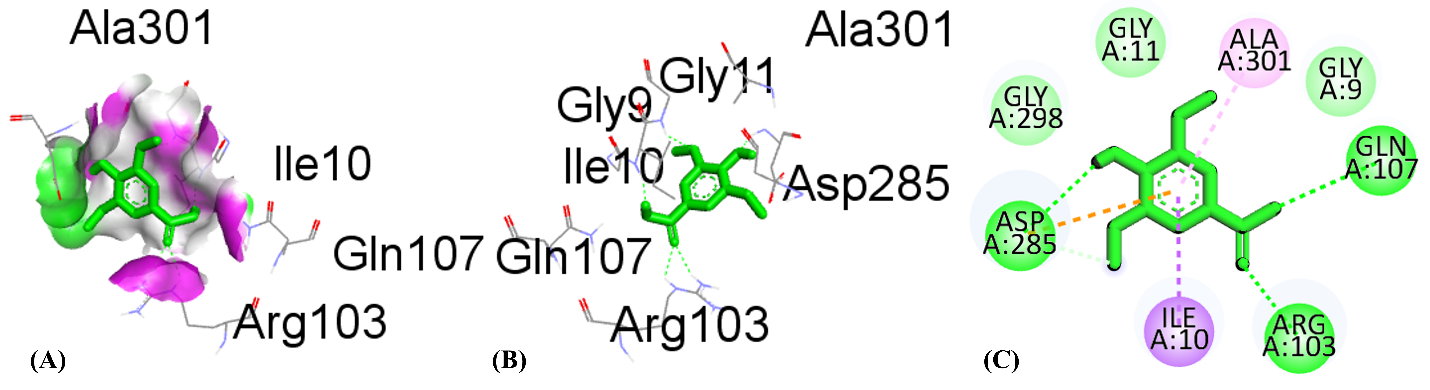


Figure S 4 : (A) hydrogen surface (B) 3D and (C) 2D interaction of syringic acid in complex with 3RP8


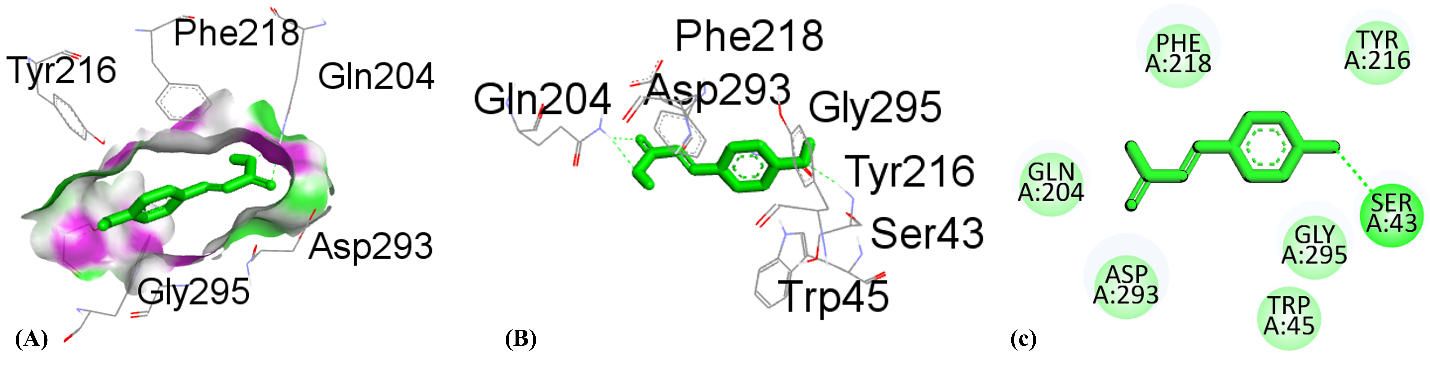


Figure S 5 : (A) hydrogen surface (B) 3D and (C) 2D interaction of P-comaric acid in complex with 3RP8


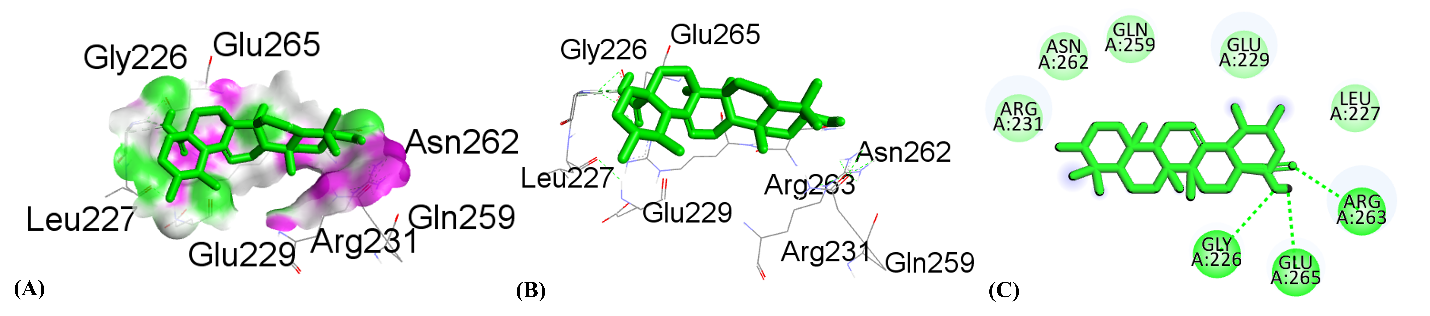


Figure S 6 : (A) hydrogen surface (B) 3D and (C) 2D interaction of ursolic acid in complex with 3RP8


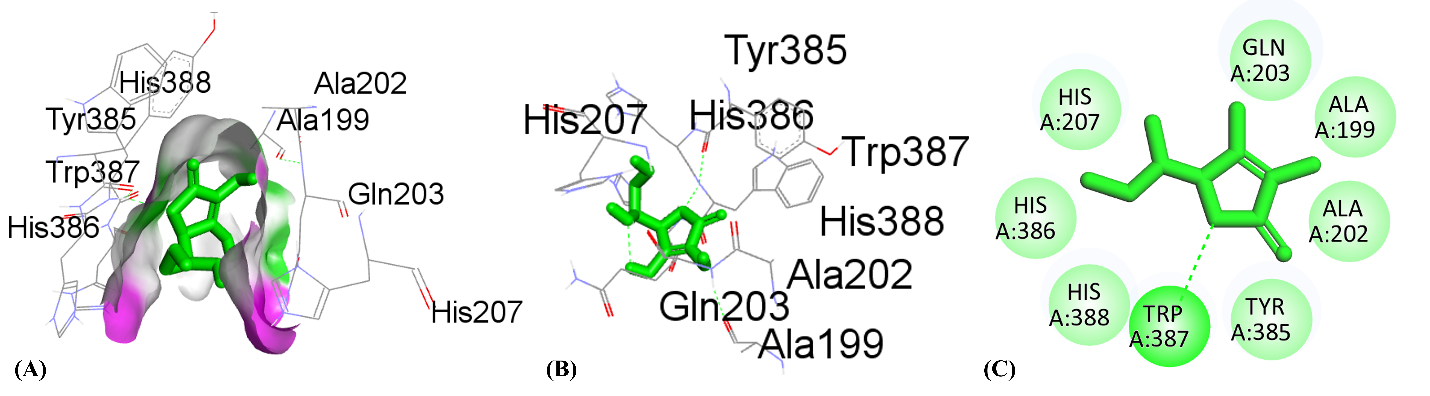


Figure S 7 : (A) hydrogen surface (B) 3D and (C) 2D interaction of ascorbic acid

in complex with 5IKQ


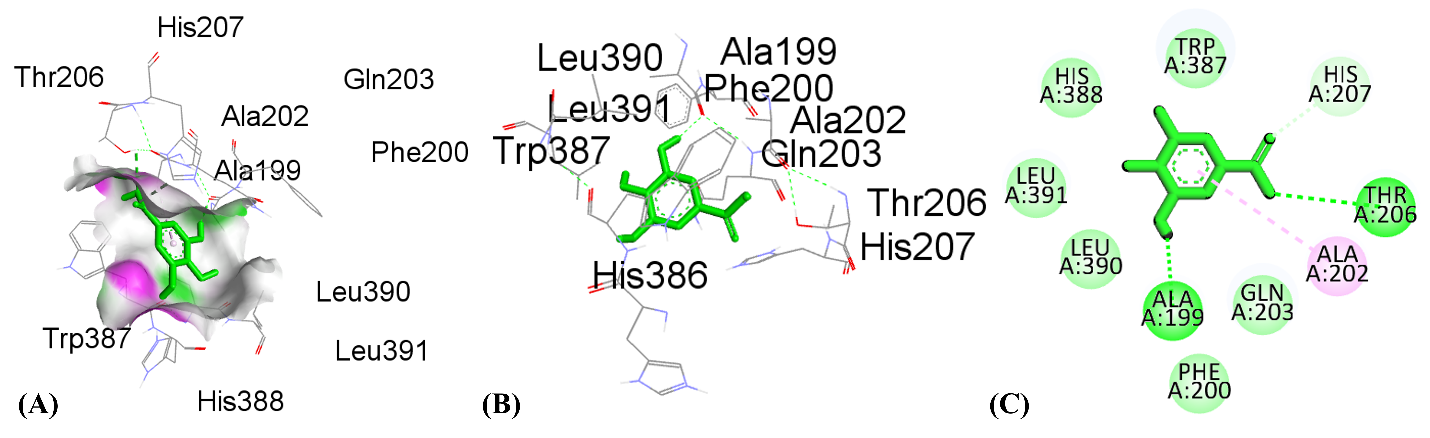


Figure S 8: (A) hydrogen surface (B) 3D and (C) 2D interaction of galic acid

in complex with 5IKQ


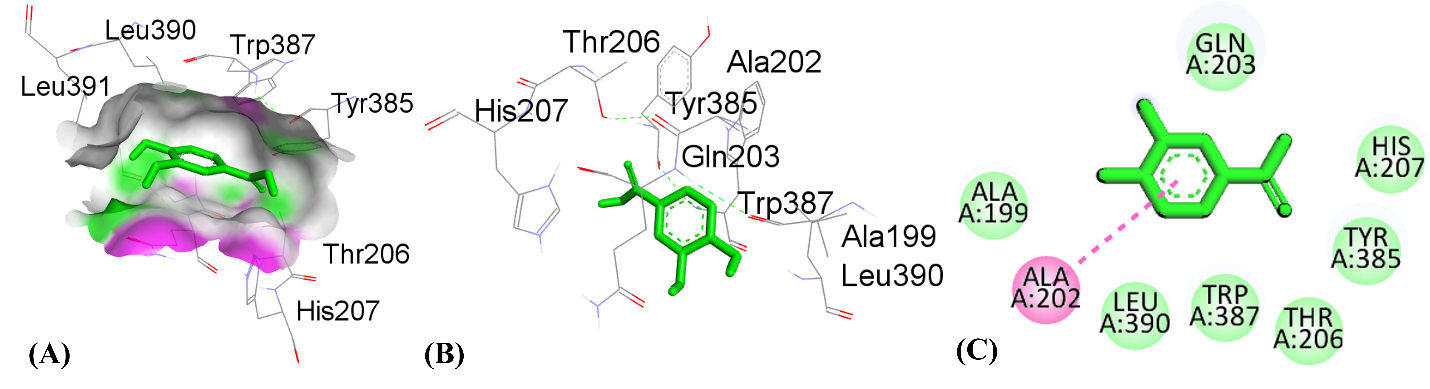


Figure S 9 : (A) hydrogen surface (B) 3D and (C) 2D interaction of 3-4-Dihydroxybenzoic acid

in complex with 5IKQ


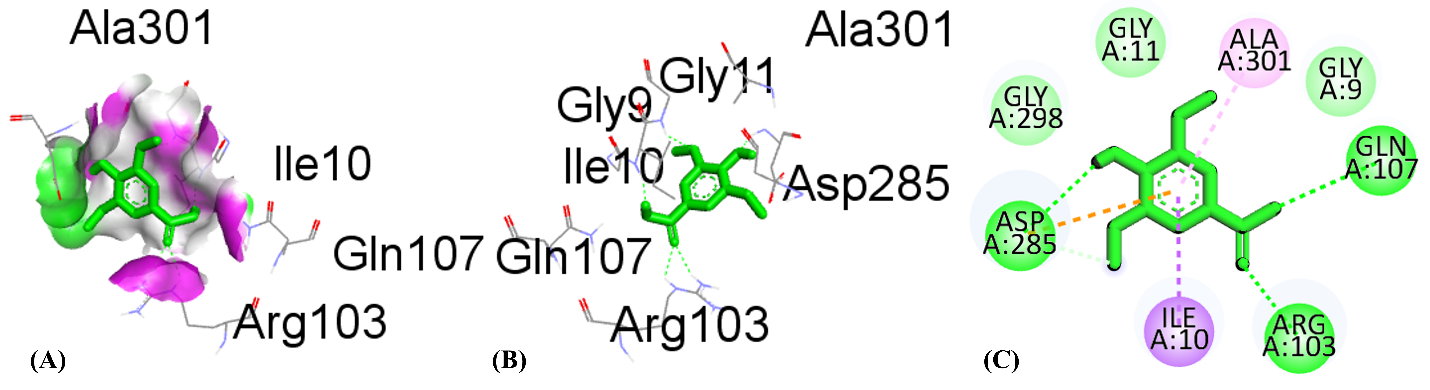


Figure S 10 : (A) hydrogen surface (B) 3D and (C) 2D interaction of syringic acid

in complex with 5IKQ


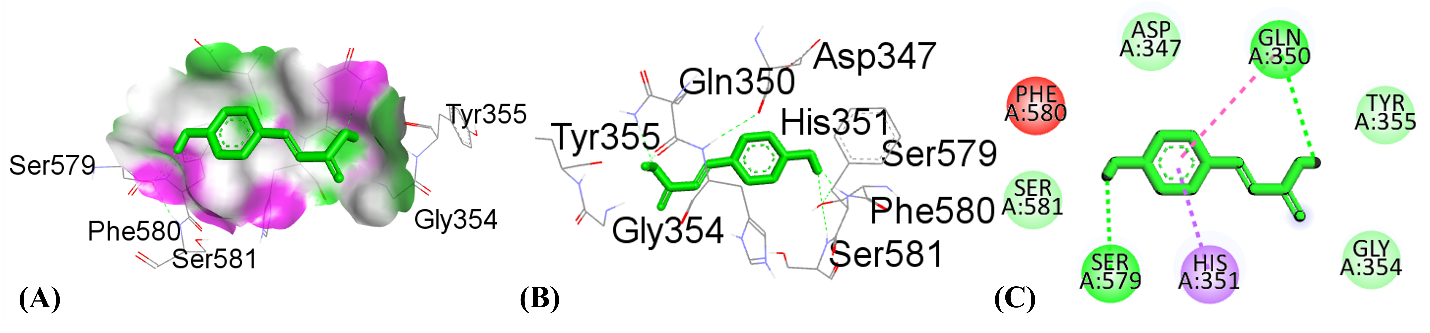


Figure S 11 : (A) hydrogen surface (B) 3D and (C) 2D interaction of P-comaric acid

in complex with 5IKQ


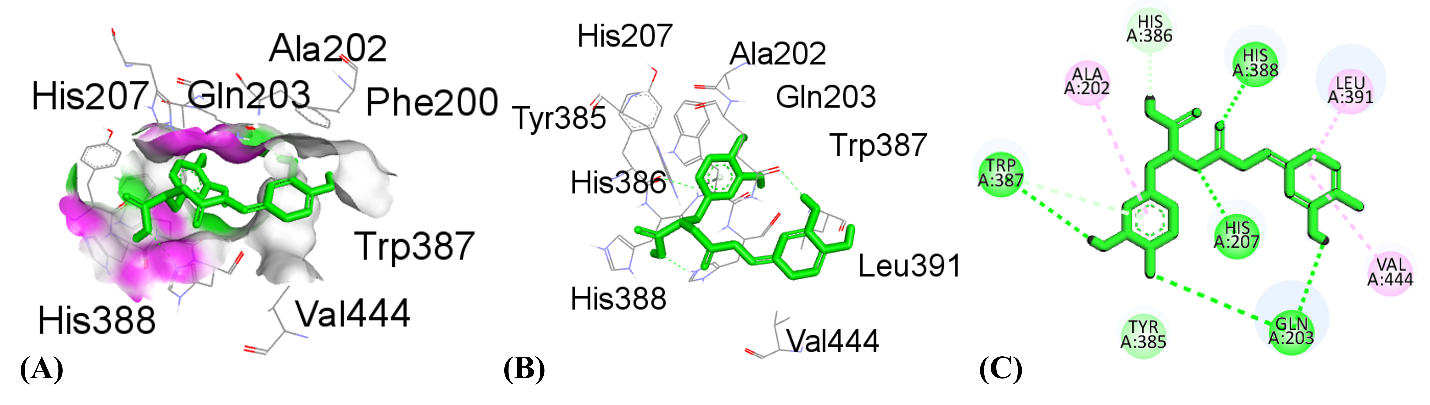


Figure S 12 : (A) hydrogen surface (B) 3D and (C) 2D interaction of rosmarinic acid

in complex with 5IKQ
